# The need for *operando* modelling of ^27^Al NMR in zeolites: the effect of temperature, topology and water†

**DOI:** 10.1039/d3sc02492j

**Published:** 2023-08-03

**Authors:** Chen Lei, Andreas Erlebach, Federico Brivio, Lukáš Grajciar, Zdeněk Tošner, Christopher J. Heard, Petr Nachtigall

**Affiliations:** a Department of Physical and Macromolecular Chemistry, Faculty of Science, Charles University in Prague 128 43 Prague 2 Czech Republic heardc@natur.cuni.cz

## Abstract

Solid state (ss-) ^27^Al NMR is one of the most valuable tools for the experimental characterization of zeolites, owing to its high sensitivity and the detailed structural information which can be extracted from the spectra. Unfortunately, the interpretation of ss-NMR is complex and the determination of aluminum distributions remains generally unfeasible. As a result, computational modelling of ^27^Al ss-NMR spectra has grown increasingly popular as a means to support experimental characterization. However, a number of simplifying assumptions are commonly made in NMR modelling, several of which are not fully justified. In this work, we systematically evaluate the effects of various common models on the prediction of ^27^Al NMR chemical shifts in zeolites CHA and MOR. We demonstrate the necessity of *operando* modelling; in particular, taking into account the effects of water loading, temperature and the character of the charge-compensating cation. We observe that conclusions drawn from simple, high symmetry model systems such as CHA do not transfer well to more complex zeolites and can lead to qualitatively wrong interpretations of peak positions, Al assignment and even the number of signals. We use machine learning regression to develop a simple yet robust relationship between chemical shift and local structural parameters in Al-zeolites. This work highlights the need for sophisticated models and high-quality sampling in the field of NMR modelling and provides correlations which allow for the accurate prediction of chemical shifts from dynamical simulations.

## Abstract 2

*Operando* modelling of ^27^Al NMR in zeolites, showing the importance of hydration and dynamics in reproducing experimental data. Machine learning analysis obtains a simple correlation of chemical shielding which predicts chemical shifts accurately.

## Graphical abstract

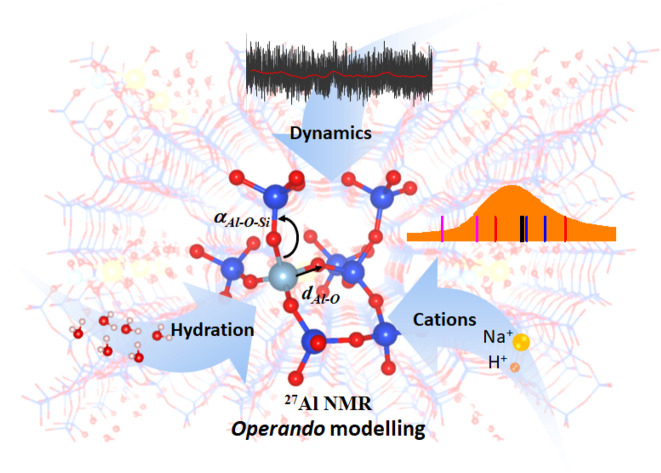

## Introduction

For decades we have been trying to understand chemical processes at the molecular level. Achieving such a goal requires systematic improvements in the models used to describe the processes in terms of atomistic details: knowledge of the structure of the catalyst active site and understanding the mechanisms of individual reaction steps. Such conceptual understanding is being built from the wealth of both experimental and computational contributions, particularly from the studies where they strongly overlap. Due to the enormous computational expense of using accurate models that describe reactive events at *operando* conditions,^1^ the best synergy between experiment and theory has been achieved for “idealized” situations: using lower pressure (only the minimum number of reacting molecules present), lower temperature, and a simplified chemical composition of the reaction mixture. While the experimental studies of the processes at *operando* conditions are well established, computational counterpart studies are in general lagging behind.

The detailed knowledge of aluminium distribution and their local environments in zeolites is among the grand challenges in zeolite science today.^2–5^ Neither the distribution of individual Al atoms among crystallographically independent framework sites nor the relative positions of two framework Al atoms can be obtained from experiments in general (except in specific cases such as Al pairs separated by just a single SiO_4/2_ tetrahedron). It is generally accepted that solid-state nuclear magnetic resonance (ssNMR)^6,7^ is the most promising technique to deliver critical information about framework aluminium and by extension, the catalytic properties.^8–15,17^ However, the interpretation of spectra are mostly based on models that rely on numerous assumptions which are not sufficiently justified.^16,18,19^ The use of static, athermal calculations, derived from local structure optimizations allows for highly accurate solutions of the Schrödinger equation, in which exchange and dispersion contributions are properly considered. However, these popular methods are unable to capture the effects of dynamic sampling over many microstates and generally require simplification of the model with respect to experiment. In general, computational models often fail to take into account various aspects of experimental conditions, including: (i) idealized chemical composition, mostly neglecting water and often neglecting even charge-compensating cations, (ii) temperature and entropy effects are not considered at all or they are severely approximated and (iii) time-averaged properties are substituted by single value obtained for the minimum on the potential energy surface (PES). While such an approach helps us to build the conceptual understanding of the problem, at the same time, it limits the space within which our understanding and chemical intuition is formed.

The experimental solid-state NMR spectra of ^27^Al in zeolites are typically obtained for zeolite samples that are exposed to ambient conditions before their NMR spectra are taken. Such zeolite samples contain some amount of water that is readily adsorbed in the vicinity of extra-framework cations when zeolites are exposed to the air. The NMR spectra are acquired over time in the order of μs. It follows that the water affects NMR characteristics: first, it may decrease the coordination of the cation with framework oxygen atoms adjacent to Al, second, it may form additional hydrogen bonding interactions with the framework, third, it may interact as a Lewis acid directly with Al sites, changing the coordination environment around Al centres. These effects were not systematically investigated so far except some early studies^20,21^ showing for MCM-58 that models considering different extent of hydration provides qualitatively different ordering of T1–T4 sites.

**Fig. 1:**
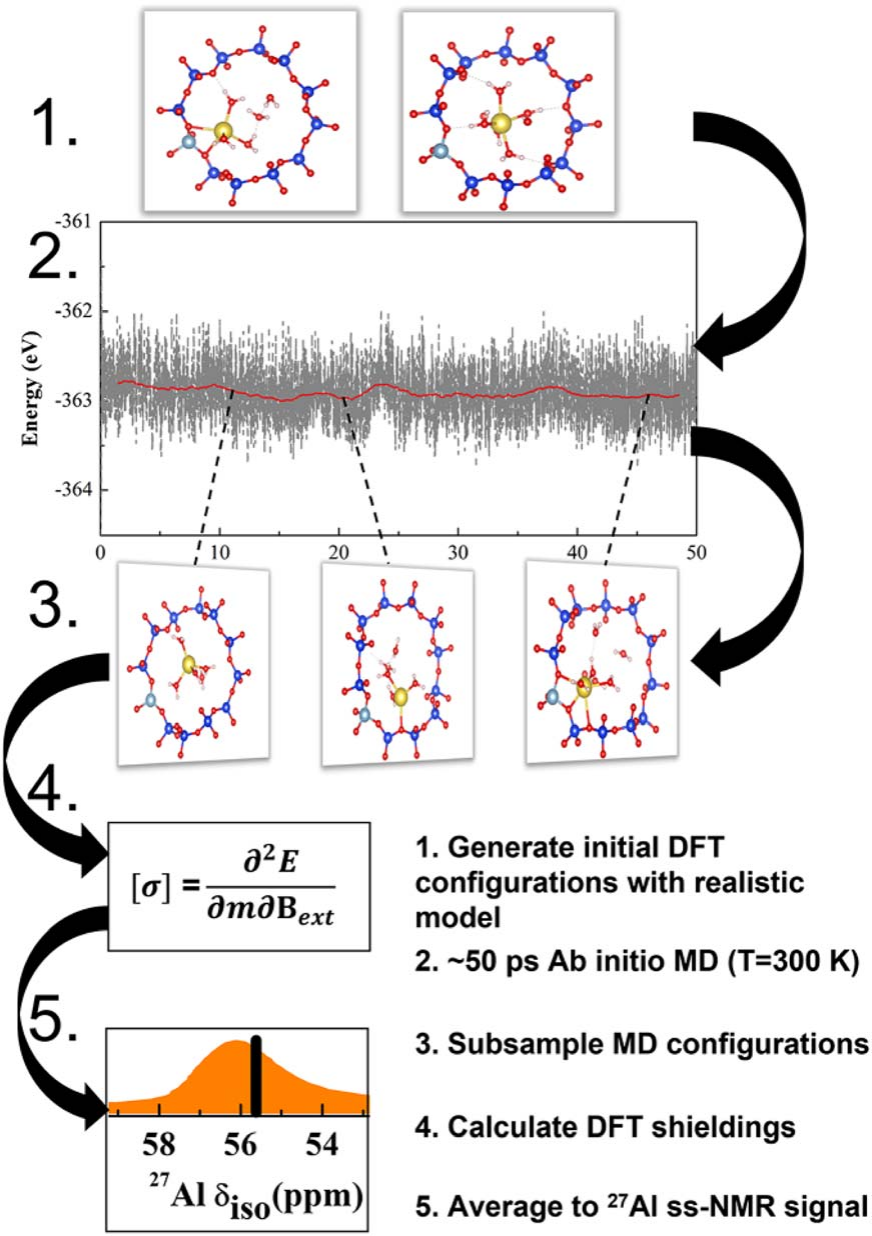
The scheme of *operando* modeling of ^27^Al ssNMR for zeolites. Such *operando* model provides reliable data to understand structure–chemical shift correlation as well as effects on ssNMR spectra, including zeolite topology and composition, hydration and thermal motion.

The NMR characteristics depend on the electron density on the nucleus, which depends in turn on the geometry in the close vicinity of Al. This is determined by extra-framework cations, their hydration and thermal motions, in addition to the zeolite topology. The most realistic model for the NMR characteristics should therefore be based on tensors averaged over the course of MD trajectory performed for a sufficiently long time upon a sample with a realistic composition (Fig. 1).^22,23^ Temperature effects and vibrational averaging of chemical shifts have been investigated for organic molecules and molecular crystals, showing good agreement with experimental data.^24–27^ It follows that the *operando* model^1^ for zeolite NMR must have a chemical composition as close to the experimental sample as possible, including Si/Al ratio, extra-framework cations and degree of hydration. Such *operando* modelling is computationally demanding and to the best of our knowledge it has not performed for ^27^Al NMR in zeolites, save for the work from van Speybroeck *et al.*;^28^ instead static and rather simplistic models (*e.g.*, models employing a continuous background charge replacing the extra-framework cation) were mostly used.^16,19,29,30^ It is the primary goal of this manuscript to establish the reliability of such models and devise a robust computational protocol for accurate and efficient NMR calculations. On examples of CHA and MOR zeolites with various Si/Al ratios it is shown that position of the chemical shift maxima depends not only on the topology of the zeolite framework but also on the type of charge compensating cation of (AlO_4/2_)^−^ tetrahedron, including level of hydration of the particular cation. It is shown that a single framework Al can show two distinctly different chemical shifts if charge compensated by cations in different channels, or if the level of hydration, or available void space, allows for hydration in one channel but not in the other.

A major step towards better understanding of ^27^Al ssNMR has been brought recently in a systematic investigation by Holzinger *et al.*,^31^ focusing on several important model parameters including the choice of exchange–correlation functional, basis set, unit cell size and the type of cluster and periodic models adopted. All calculations were based on the models which do not explicitly consider the extra framework cation. Thus, the framework charge remained negative without any compensation (cluster models) or it was charge-compensated by a positive background charge (periodic models). Such models are often denoted as “background-charge” (bgc) models. Considering all important conclusions drawn by Holzinger *et al.*, our goal is to go beyond the bgc model and towards an *operando* model, mimicking the experimental situation as closely as possible, and to show that this is necessary.

## Models and methods

### Zeolite models

Only the most important models and method characteristics are described in the text, more details can be found in ESI.† Calculations were performed with various models of MOR and CHA zeolites. Models are characterized by 3 key parameters: (i) character of the charge-compensating ion (Na^+^, H^+^ or bgc); (ii) degree of hydration; and (iii) temperature. The adopted notation ABC(*n*)|M|*x*w is defined as follows: ABC denotes zeolite framework and *n* = Si/Al is the silicon–aluminum ratio; M is the charge-compensating cation and *x* is the number of water molecules explicitly considered in the unit cell (UC). Individual types of models are shown in Fig. S3.† Models relying on background charge are denoted bgc|0w and no water is explicitly considered (only static calculations and “non-averaged” *δ* values are reported). Models representing completely dehydrated Na- and H-form of zeolites are denoted Na|0w and H|0w, respectively. Models representing hydrated Na-zeolite and H-zeolites are denoted Na|*x*w and H|*x*w, respectively. For more details about the models see Fig. S3.† Unless stated otherwise, a water density of 1 g cm^−3^ in zeolite channels and cavities was used for zeolite hydration. This corresponds to 6 and 5 water molecules in the UC for MOR and CHA, respectively.

The effect of temperature is considered by means of *ab initio* molecular dynamics (AIMD) simulation (order of tens of ps) and averaging the NMR tensors for 100–500 structures selected equidistantly along the MD trajectory. Corresponding chemical shifts and models are denoted as *δ* and ABC(*n*)|M|*x*w, respectively. These quantities will be referred to as “dynamic” as they are based on MD simulations and averages of shielding tensors obtained for structures populated at a given temperature. Unless stated otherwise, UC cells consisting of 12 and 24 T-atoms were used for CHA and MOR zeolites, respectively (the effect of the UC size has been considered, in terms of cell optimization and supercell size, and is described herein). The structures and numbering schemes of both zeolites are shown in Fig. S2.†

## Methods

Geometry optimization as well as AIMD simulations (Born–Oppenheimer molecular dynamics, BOMD) were performed at the DFT level with the Vienna *ab initio* simulation package (VASP) with the projector-augmented wave (PAW) pseudopotentials.^32–35^ The energy cut-off was set to 700 eV, the wave function convergence to 10^−6^ eV, and forces convergence criteria for geometry optimization to 0.01 eV Å^−1^. The Brillouin zone was sampled using a 1 × 1 × 2 *k*-point grid for MOR, which was deemed *via* convergence tests to be sufficient, and the Gamma point was used for CHA. We used the generalized gradient approximation (GGA) Perdew–Burke–Ernzerhof (PBE) functional^36^ and included the description of dispersion forces according to the Becke-Johnson (BJ) damping model^37^ (PBE + D3). While PBE functional is known for bulk water over-structuring,^38^ it is comparable to other GGA functionals for the description of water clusters and the quality of description is improved by the BJ damping correction.^37^ The structures are optimized with cell constraints fixed to the optimal value for one model within the zeolite class. For example, the fixed cell of CHA uses the optimized parameters of the CHA(11)|Na|5w system, which is due to two reasons: (i) the optimized CHA(11)|Na|5w model has a similar cell volume to the model from IZA and is thus close to both experimental and other computational studies; (ii) a systematic variation of the volume of CHA showed that the slight change that occurs upon optimization (<20 A^3^) does not dramatically affect the ^27^Al chemical shift (<2 ppm shift) (Fig. 4). This is discussed thoroughly in the Discussion section below. Based on the CHA tests, the model from the IZA database is used for MOR.

The AIMD trajectory simulations were performed with an energy cut-off for the plane-waves of 400 eV at 300 K within the canonical NVT ensemble, using a 0.5 fs time step and a Nosé–Hoover thermostat. Well-equilibrated trajectories of between 20 and 50 ps were carried out for each simulated system.

The NMR tensors were calculated with GIPAW (gauge-including projector augmented-wave method) as implemented in the CASTEP program suite.^39,40^ We used the PBE functional revised for solids (PBEsol)^41^ for NMR calculations. An energy cutoff of 700 eV and *k*-point sampling set of 2 × 2 × 2 for CHA single cell and 1 × 1 × 2 for MOR single cell were used to converge the total energy below a threshold of 10^−8^ eV per atom.

To relate the experimental chemical shift *δ*^exp^ and calculated chemical shielding *σ*^cal^, we used a linear regression based on four experimental values obtained for Al(acac)_3_ and three hydrated zeolites in acidic form, considering both tetrahedral and octahedral aluminium (see Fig. S4 and further related discussion in the ESI for more details†):

1 *δ*^exp^ = 1.116*σ*^cal^ + 609.72

The deviation of the slope from 1 must be interpreted as a systematic error due to the model and/or method used in the calculations of the chemical shielding tensor but partially also to the uncertainties in the assignment of a particular structure to a particular signal. Nonetheless, the slope obtained for the correlation (based on relatively few data) is within the precision obtained previously for other elements^42^ and the −1.12 value reported for ^29^Si.^43^

A linear regression was adopted to describe the relationship between (physically interpretable) geometrical descriptors and ^27^Al chemical shielding, employing structural features selected by the least absolute shrinkage and selection operator (LASSO). LASSO is a regularized linear regression method that limits the model complexity to the most relevant input features in order to avoid overfitting. Here, the LASSO regression (see Table S1, Fig. S1† and Fig. 5 for details)^44^ used twelve initial input features and more than 1400 DFT values from MD trajectories with various models of MOR and CHA. Calculation of structural features and LASSO regression used the python packages Atomic Simulation Environment (ASE) and Scikit-learn, respectively.^45^

## Results

### Critical comparison and validation of models

**Fig. 2:**
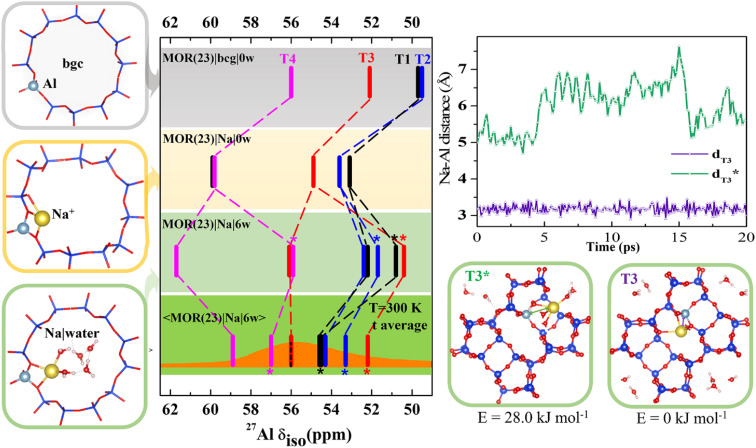
Chemical shifts calculated for MOR (Si/Al = 23) with various models; the bgc model is shown in a gray frame and corresponding *δ* values are shown on the same color background. The completely dehydrated and hydrated models with Na^+^ charge-compensating cations are shown in yellow and green frames, respectively. Static *δ* and dynamic average 〈*δ*〉 values of the Na|MOR(23)|6w model are shown on a lighter and darker green background, respectively, with the experimental range of MAS-NMR (from ref. 13 and 14) overlaid in orange and the value of the peak maximum from MQMAS NMR, for which the effect of quadrupolar coupling is removed,^6^ as a dotted line.^14^ Competition between fully and partially solvated cations is given for Al in T3 position. It is shown that solvation of Na^+^ cation residing on the intersection of interconnecting cavity and 12R channel (denoted T3*) and in the limiting 8R channel along [001] (denoted T3) is rather different and is reflected by a large difference in Na–Al distances and a 4 ppm difference in chemical shifts. Note: in MOR(23)|Na|6w the fully hydrated Na^+^ cation is distinguished with an asterisk(more details are shown in Fig. S6†); there are two T1 configurations in MOR(23)|Na|0w within 1.0 kJ mol^−1^ of each other, so both are shown (shown in Fig. S5†).

To gain an insight into the importance of individual model parameters for calculated chemical shifts we performed calculations with different models of increasing complexity. Models are chosen to allow for discussion of individual parameters separately: effects of explicit cation, cation solvation and of temperature. Therefore, chemical shifts were calculated for each of four distinct positions of Al in MOR with Si/Al = 23 (Fig. 2 and Table S2†). Four models were considered: (i) the simplistic background charge MOR(23)|bgc|0w model (without an explicit extra-framework cation), (ii) the MOR(23)|Na|0w model with explicit Na^+^ extra-framework cation at the position obtained by geometry optimization of an exhaustive set of initial configurations, (iii) the MOR(23)|Na|6w model with Na^+^ extra-framework cation solvated by 6 water molecules at the geometry of minimum-energy structure obtained by geometry optimization, and (iv) the 〈MOR(23)|Na|6w〉 model where the chemical shielding tensor is averaged along the MD trajectory obtained for a solvated Na^+^ cation. While the Al environment used for chemical shift calculations is represented by a single structure (corresponding to 0 K) for models (i)–(iii), the chemical shift calculated for the last model corresponds to Al environment represented by an MD trajectory at 300 K. Chemical shifts calculated with the MOR(23)|bgc|0w model are in the range of 50–56 ppm (Fig. 2). The chemical shift obtained for Al in T4 is in good agreement with experimental results, while the chemical shifts found for Al in T1 and T2 positions are too small (<50 ppm) compared with the experimental value around 56 ppm.^14^ The advantage of the simple background-charge model is a relatively symmetrical environment of framework Al (not perturbed by the presence of a cation) that is reflected by low *C*_q_ values (around 3 MHz, Table S2†). However, there are numerous disadvantages of this model that are revealed by comparison with the results obtained with more sophisticated models.

The effect of the explicit charge-compensating cation (with respect to background charge) is clearly visible from the comparison of chemical shifts obtained with MOR(23)|bgc|0w and MOR(23)|Na|0w models (Fig. 2). Na^+^ cations are closely attached to framework oxygen atoms of (AlO_4/2_)^−^ tetrahedra (Fig. S5†), which thus affect the Al–O–Si angles (and thus the electron density on the Al nucleus) and introduce additional anisotropy on framework Al. Consequently, the presence of Na^+^ leads to increased chemical shifts by 3–4 ppm towards experimentally observed values and to increased anisotropy, leading to larger *C*_q_ values (above 5 MHz). Moreover, the presence of Na^+^ results also in qualitative changes: (i) chemical shifts for Al in individual T sites are in a different order to those obtained with the bgc model, showing that the interpretation of experimental data depends on the model used. (ii) There are two signals from Al in the T1 site. Due to the complex structure of MOR, Al in some T positions can be charge-compensated by Na^+^ cations approaching (AlO_4/2_)^−^ tetrahedron from different channels (Fig. S5†), which may result in rather different chemical shifts. If such structures have similar energies, they may both be observed in the NMR spectra when they do not interchange on the time scale of the NMR measurements. Two distinctly different values of chemical shifts due to the single framework Al atom will be denoted “δ-doublets”. Within the MOR(23)|Na|0w model the δ-doublet is reported for Al in T1, showing shifts at 53 and 60 ppm for Na^+^ compensating Al from the main channel and from the limiting 8R channel along [0 0 1], respectively. For Al in other T-sites there is either a larger energy difference between the most stable Na^+^ and other Na^+^ sites (T3 and T4), or the δ-doublets are close together (T2) (Fig. S5†). Thus, only one value of *δ* is reported in Fig. 2 for those Al positions.

The effect of cation solvation is apparent from the comparison of chemical shifts obtained with MOR(23)|Na|0w and MOR(23)|Na|6w models (Fig. 2). Interaction of Na^+^ with water weakens the interaction of Na^+^ with framework oxygen atoms of the (AlO_4/2_)^−^ tetrahedron, relaxing Al–O–Si angles and partially decreasing the anisotropy of Al environment (*C*_q_ < 5 MHz, Table S2†). There are two distinctly different solvation modes of Na^+^ in MOR channels: (i) partial Na^+^ solvation where the Na^+^ first coordination shell consists of 2–3 framework oxygen atoms and of 3–4 water molecules (left column in Fig. S6†). (ii) Full Na^+^ solvation, where the Na^+^ cation moves towards the center of the channel system, loses coordination with framework oxygen atoms and its first coordination shell consists solely of water molecules; there are 4–6 water molecules in the first coordination shell, depending on the void space available (right column in Fig. S6†). The energy difference between partially and fully solvated Na^+^ structures is above 20 kJ mol^−1^ in favor of partially solvated structures, except the case of Al in T1, where the fully solvated structure is only 7 kJ mol^−1^ higher than corresponding partially solvated structure (Fig. S5†). Therefore, chemical shifts of both structures for Al in T1 are reported in Fig. 2 together with chemical shifts of partially solvated structures for other Al positions. The cation solvation leads to a large spread of calculated chemical shifts (between 51 and 62 ppm) and there are also qualitative changes in relative peak positions due to Na^+^ solvation.

The most complex model considered here is the dynamic model of solvated sodium form of MOR, the 〈MOR(23)|Na|6w〉 model. Instead of using a single structure, the one with minimum energy and corresponding to 0 K, the chemical shielding tensor is calculated as an average along the MD trajectory (represented by 100 or more equidistant snapshots). Thus, the sampling is over the structures populated at given temperature (300 K used in this study). The consideration of temperature-induced dynamics does affect *δ* values significantly, changes from −5 to +4 ppm are reported in Fig. 2. Calculated chemical shifts are in the range of 52–59 ppm, in excellent agreement with experiment. The existence of both partially and fully solvated Na^+^ structures discussed for static 0 K model above also holds for the dynamic 300 K model: *δ* values for both partially and fully solvated structures are reported in Fig. 2. The differences between partially and fully solvated Na^+^ are shown for Al in T3 in Fig. 2. When the Na^+^ cation charge-compensates Al from the space-limited 8R channel, there is no space for water to fully solvate such a cation; instead Na^+^ sits in the same position throughout the MD simulation (Na–Al distance reported in Fig. 2) and chemical shift remains unchanged. In contrast, Na^+^ compensating Al in T3 from the opposite side (main channel window) can become fully solvated and Na–Al distances along the MD trajectory show that partially and fully solvated situations interchange during the MD simulations. Na^+^ in the limiting 8R channel along [001] results in *δ* = 56 ppm while Na^+^ in the main channel gives *δ* = 52 ppm. MD simulations indicate that the differences in average energies *U* obtained for both structures are less than 30 kJ mol^−1^ (Fig. S7†). The existence of framework Al showing δ-doublets is not limited to Al in T3 site and 〈MOR(23)|Na|6w〉 model; it is rather general and corresponding situations are shown in Fig. 2, where *δ* values obtained for fully solvated cations are marked with a star.

Results reported in Fig. 2 clearly show that relative values of chemical shifts obtained for Al in different T-sites depend qualitatively on the model complexity. We conclude that reliable interpretation of experimental spectra (an assignment of framework Al positions in particular) can only be obtained with models considering both experimentally relevant amounts of water and temperature fluctuations, as is demonstrated with the 〈MOR(23)|Na|6w〉 model.

### Quantification of Si/Al ratio, cation type and temperature effects in MOR

Using the dynamic model we have investigated the following effects (Fig. S8†): (i) Si/Al ratio (and corresponding extra-framework cation concentration), (ii) type of cation – Na^+^*vs.* H^+^, and (iii) temperature. Models of MOR with the Si/Al = 23, 11, and 7 (consisting of 3, 2, and 1 Al in UC, respectively) were used. Both 〈MOR(11)|Na|5w〉 and 〈MOR(7)|Na|5w〉 models give very similar 〈*δ*〉 values as the 〈MOR(23)|Na|6w〉 model for signals corresponding to partially solvated structures. Spectra obtained for MOR with lower Si/Al become less complex, since there is not enough water to fully solvate all Na^+^ cations, *i.e.* only partial solvation of the cations is possible. For MOR in H-form, all three protons may be solvated, but dynamic simulations show that a rapid equilibrium between solvated and unsolvated protons is established in the T1 and T3 sites, leading to an averaging of chemical shifts between configurations and thus a qualitatively different order of peaks from the results found for Na-form of MOR. Finally, Fig. S8† shows that temperature effects are always important for hydrated zeolites; they are less important for a completely dry zeolite as seen by comparison between MOR(23)|Na|0w *vs.* 〈MOR(23)|Na|0w〉 models.

### The role of zeolite topology – the simple zeolite CHA

A very strong dependence of calculated chemical shifts on the model complexity clearly demonstrated above contradicts some conclusions drawn in previous computational studies. Various effects including suitability of exchange–correlation functional, Si/Al ratio, supercell or the nature of the extra-framework cations have been investigated, typically for zeolites with just a single T-site or with just a 1-D channel system.^28,30,31^ Before in depth discussion of some discrepancies, we present the results obtained for CHA zeolite, a commonly used zeolite in both experimental and computational investigations, which has high symmetry and only one symmetry inequivalent T-site.

**Fig. 3:**
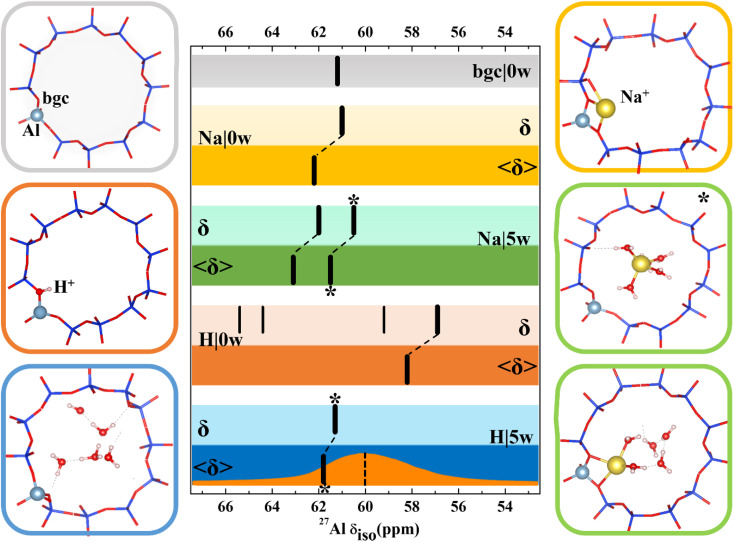
Models used for ^27^Al NMR calculations and chemical shifts calculated for CHA(11) zeolite with constant volume (*V* = 796.35 Å^3^, optimized for the CHA(11)|Na|5w model). Chemical shifts are denoted by black bars. The bgc model is shown in a gray frame and corresponding *δ* value is shown on the same color background. For dehydrated Na-CHA, hydrated Na-CHA, dehydrated H-CHA and hydrated H-CHA(*), *δ* values are shown on a lighter background for static *δ* and a darker background for averaged 〈*δ*〉 values, in the colors yellow, green, orange and blue, respectively. In the CHA(11)|Na|5w model, the fully solvated Na^+^ cation is distinguished with an asterisk. MD averaging of *δ* was performed only for the most stable BAS configuration. The orange plot in the bottom row shows the experimental MAS NMR,^5^ while the dotted line refers to the peak maximum of the MQMAS spectrum from ref. 15.

With the exception of dehydrated H-CHA, chemical shifts calculated with all CHA models fall within 2 ppm and the agreement is even better when we compare results obtained with simpler static models (Fig. 3 and Table S3†). There is a small and systematic effect of temperature showing up to 2 ppm increased values of 〈*δ*〉 with respect to corresponding static *δ* values. The anomalously small values of chemical shifts found for dehydrated H-CHA(11) (Fig. 3) are due to the larger average Al–O bond length and *α*_Al–O–Si_ angle at the Brønsted site, which is in turn due to the lack of proton solvation (Table S5†).

Results for CHA indicates that the model sophistication does not affect the chemical shift values significantly and that the position of charge-compensating cation with respect to framework Al does not influence the aluminum local environment enough to lead to appreciable changes of chemical shifts. The only exception is the H|0w model, which describes dehydrated H-CHA and gives both *δ* and 〈*δ*〉 values about 5 ppm lower than other models. However, this is the “invisible” peak known to experiment. The presence of a proton in the BAS site results in high anisotropy of the aluminum environment (reflected by a large *C*_q_ value, Table S3†). The value of *C*_q_ is calculated to be around 20 MHz, which is in accord with the experimental observation that the BAS site Al is not seen under dehydrated conditions.^46–48^ Upon proton solvation in the CHA cage (H|5w model), calculated chemical shifts are in line with the results obtained with other models and the *C*_q_ values decreases to 1.4 MHz (*P*_q_ = 1.6 MHz), thus making the peak visible. By comparison, the experimental quadrupolar product *P*_q_, *C*_q_(1 + *η*^2^/3)^1/2^ is found to be 1–2 MHz for a BAS site in MFI.^17^

Inspection of MD trajectories obtained for the 〈CHA(11)|Na|5w〉 model shows that in addition to fully solvated Na^+^, a partially solvated Na^+^ mode occurs (Fig. 3), which is about 10 kJ mol^−1^ higher in energy than fully solvated Na^+^ (Table S3†), from static calculations. While the Na–Al distance for the two modes differs by as much as 2 Å, the corresponding averaged 〈*δ*〉 values are within 1.6 ppm. Corresponding static calculations (optimized structure for fully and partially solvated Na^+^) also give a difference in *δ* of 1.6 ppm. Additional MD simulations indicate that the average energies *E* obtained for fully and partially solvated Na^+^ are very similar (Fig. S9†), thus, the chemical shielding tensor should be averaged over a sufficiently long simulation to properly sample both types of Na^+^ solvation modes at their proper occupation probabilities. Over the simulations performed here (50 ps duration), the average shift is 62.4 ppm. The calculated *C*_q_ values reflect the higher symmetry of the Al environment upon solvation, decreasing from 6.7 MHz (*P*_q_ = 7.0 MHz) for dehydrated Na-CHA to 3.2 MHz (*P*_q_ = 3.2 MHz) for the fully solvated configuration. This overestimates, but agrees with the trend from experiment, which shows that hydration leads to a *P*_q_ decrease from 4.2 MHz to 1.8 MHz.^49^

**Fig. 4:**
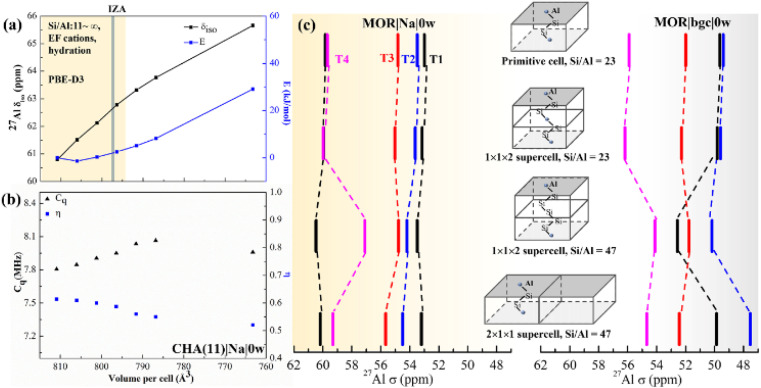
The effect of the UC size and parameters on calculated chemical shifts. (a) The calculated *δ* as a function of isotropic cell volume variation in CHA(11)|Na|0w along with the relative energy variation. The yellow background covers the full volume range obtained at the PBE-D3 level for CHA with Si/Al from ∞ to 11, including all charge-compensation models and hydration degrees (see also Tables S7 and S8†). The grey bar denotes the volume from the IZA database; (b) changes of *C*_q_ and *η* with volume for the same zeolite model. (c) Calculated ^27^Al chemical shifts in MOR using various supercells, highlighting the importance of local structure and Si/Al ratio over supercell size.

In summary, chemical shifts calculated for CHA(11) are not significantly affected by the model parameters. Only a small decrease of *δ* and *δ* with the UC volume increase is found, with a variation of only around 2 ppm over the full range of optimal cell volumes considered (Fig. 4). Furthermore, for a given water loading, the range of volumes is smaller still, and thus the variation of the chemical shielding is less than 2 ppm (Table S7†). The effect of temperature, obtained by averaging of shielding tensors along the MD trajectory, gives up to 2 ppm increase of the chemical shift. While the effect of H^+^ solvation is significant, the solvation of Na^+^ charge-compensating cation does not have any effect, either on *δ* or 〈*δ*〉 values.

## Discussion

### Interpreting the structure–shielding relationship

A simple relationship between the ^27^Al chemical shift and the geometry of (AlO_4/2_)^−^ tetrahedron was proposed by Lippmaa for Al-rich zeolites more than three decades ago.^50^ A linear dependence of *δ* on averaged T–O–T angle is understandable, assuming that the Al–O distances do not change appreciably in zeolites.^51^ This correlation has been used in many studies, including assignment of experimental ^27^Al chemical shifts to Al in the particular framework position (based on the T–O–T angles from diffraction measurements).^31,52,53^ It has been found useful in some studies and rather inaccurate in other studies, in particular in high-silica zeolites.^19,20,53^

**Fig. 5:**
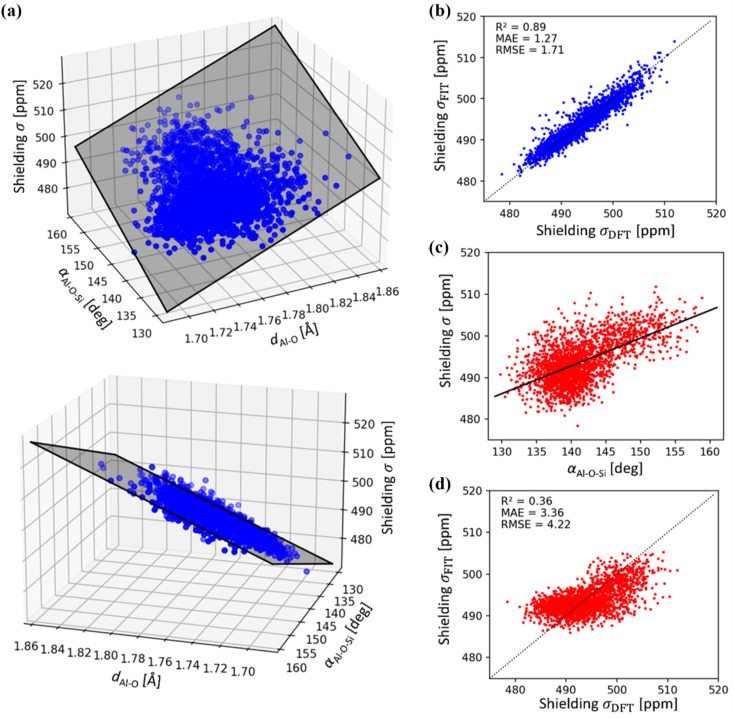
(a) ^27^Al chemical shielding *σ* as a function of average Al–O distances *d*_Al–O_ and Al–O–Si angles *α*_Al–O–Si_ of (AlO_4/2_) tetrahedra. Shaded area represents the linear regression model (see eqn (2)). (b) Correlation between LASSO linear regression model (see eqn (2)) and DFT ^27^Al chemical shielings (*R*^2^ = 0.89). (c) Correlation between ^27^Al chemical shielding (*σ*) with *α*_Al–O–Si_ as proposed by Lippmaa,^50^ using DFT reference data (d) correlation between linear regression model according to Lippmaa,^50^ and DFT ^27^Al chemical shielings, showing a weak relationship (*R*^2^ = 0.36). Note, mean absolute (MAE) and root mean squared error (RMSE) are in units of ppm.

Using a sub-sample of 1466 structures from MD simulations carried out for dehydrated and hydrated CHA in both H- and Na-forms, the correlation between chemical shielding *σ* and average Al–O–Si angle is plotted in Fig. 5. It is immediately apparent that Lippmaa's one-parameter (T–O–T angle) correlation^50^ provides only a rough correlation (*R*^2^ = 0.36), even for the simple CHA zeolite and it cannot be used for the assignment of ^27^Al NMR spectra (Fig. 5). Since the electron density on the nucleus must primarily depend on the local geometry, we have analyzed the effects of local geometrical descriptors on *σ* by LASSO regression. Here, we used LASSO for the selection of as few as possible physically interpretable features, such as bond distances and angles, to obtain a simple linear structure–*σ* correlation for detailed analysis of the MD trajectories. Out of twelve geometrical descriptors considered, only two were found to be important (Table S1 and Fig. S1†): the averaged Al–O bond lengths (*d*_Al–O_) and the averaged Al–O–Si angle (*α*_Al–O–Si_):

2 *σ* = *c*_1_*d*_Al–O_ + *c*_2_*α*_Al–O–Si_ + *c*_3_

with coefficients *c*_1_ = 180.60 ppm Å^−1^; *c*_2_ = 0.800 ppm per degree; *c*_3_ = 64.15 ppm. Eqn (2) provides significantly improved agreement with shieldings obtained from *ab initio* calculations for both zeolites tested (*R*^2^ = 0.89) and, due to its simplicity, it also provides the opportunity to understand shieldings in terms of only a few local geometrical descriptors. Furthermore, the error resulting from the use of eqn (2) is smaller than errors resulting from the simple models (Fig. 5). Nevertheless, it should be noted that this improved correlation requires averaging over many configurations *via* dynamical simulations, and thus is not a suitable tool for predicting Al positions in a zeolite from a static model. This model, based on LASSO regression, is designed to reduce errors due to overfitting, by excluding parameters which contribute least to the fit. An example of the generality of the fit can be found in ref. 54, in which a two parameter fit was found to apply also to a high silica MFI model. More accurate shielding (tensor) predictions can be achieved using, *e.g.*, Kernel Ridge Regression^54,55^ or (equivariant) neural networks.^56,57^ However, these ML methods use high-dimensional descriptors, making a physical interpretation of the structure–*σ* correlation difficult. This is in contrast to the correlation given in eqn (2) which uses simple distances and angles as input.

It is notable that individual geometrical descriptors, as well as calculated shieldings vary significantly along the MD trajectory as is shown in Fig. S10 and S11,† for CHA and MOR, respectively. Fluctuations of chemical shielding depend on the model used, and the largest are observed for Na-zeolites with water (about 20 ppm). While under hydrated conditions, Na^+^ gives larger fluctuations than H^+^, due to the larger range of partial solvation configurations, the opposite is true in the dehydrated case, where Na^+^ is more fixed than H^+^ and gives a smaller fluctuation. The thermal effects do not depend only on the zeolite framework itself but also on the volume and shape of the channel void space in the vicinity of individual Al atoms. The extent of Na^+^ solvation depends on the zeolite topology and particular position of framework Al and the resulting distance between Al and Na indirectly affects the chemical shielding (dominantly *via* changes in averaged *α*_Al–O–Si_ angles, Fig. S5, S6, Tables S5 and S6†).

### Effect of the UC size and volume

It is known, by the structure–shielding relationship obtained above and experimental studies that NMR is sensitive to the local geometrical parameters. Al substitution expands the unit cell, as the equilibrium Al–O bond length is greater than that of the Si–O bond. Therefore, we investigated the effect of UC size and volume on chemical shift and *C*_q_.

First, we optimized the UC of different models of CHA(11) (Fig. S3†). This leads to a volume change of up to 20 Å^3^ with respect to the volume of 796.35 Å^3^ obtained for solvated Na-CHA, for which the shifts were presented in Fig. 3. The volume change upon cell optimization results in only up to a 2 ppm change in chemical shift (Table S7† in bold). It is worthwhile to note that the results of the metastable protonic cases do not always follow the tendency of the volume change. For example, CHA(11)|H|0w with BAS on O_2_ (9.1 kJ mol^−1^ energy higher than the most stable case) shows around 2 ppm higher *δ* and 14 Å^3^ larger volume. This is due to the hydrogen bonding interaction between the proton in the BAS site and an adjacent oxygen atom of the framework,^58,59^ which affect the chemical shifts much more significantly than the volume change. Overall, the volume changes upon moving between models at fixed Si/Al ratio are relatively small in CHA, and their effect on the chemical shifts is also fairly small, and within the range that is due to proton location, cation solvation and thermal effects.

To systematically gauge the volume effect, we isotropically varied the UC volume for a representative model (CHA(11)|Na|0w) over the volume range from 810 to 760 Å^3^. The dehydrated Na-CHA model was chosen because it is the simplest case, avoiding the complicated effect of water configuration and the significant effect of the BAS sites in H-CHA. The selected volume range extends far below the equilibrium UC volumes for all CHA(11) models considered, which are recorded in Table S7.† Over this range, geometrical parameters, chemical shielding and chemical shifts and contribution of individual terms of eqn (2) are summarized in Table S4.† The chemical shift increases linearly from 61 to 66 ppm with decreasing UC volume within the range. This is in-line with the 10 ppm range found by Goltl *et al.*^30^ for a volume change in range of 800–880 Å^3^. However, it is important to note that we never observe a volume change of such a large magnitude for any CHA model, including those of different Si/Al ratios ranging from 11 to infinity. The increase of chemical shift is dominantly (80%) due to decreased average *α*_Al–O–Si_ angle (from 143 to 139°) and partially due to small decrease of *d*_Al–O_ distance (0.005 Å). Values of individual descriptors obtained with various models are shown in Table S5† for CHA and in Table S6† for Al in four T-sites in MOR. The range of *C*_q_ and *η* change slightly over the volume range considered, only about 0.3 MHz increase and 0.1 decrease with the volume, respectively (Fig. 4b).

The effect of the supercell and Si/Al ratio on calculated chemical shifts in Si-rich systems is shown in Fig. 4c. Shifts calculated with the MOR unit cell (24 T atoms, Si/Al = 23) are compared with those obtained with supercells repeated along the *c* and *a* lattice vectors (Si/Al = 47). Chemical shifts obtained with the model of dehydrated Na-MOR, MOR(23)|Na|0w, do not depend on the supercell, except the chemical shift for Al in T4 position calculated with (1 × 1 × 2) supercell and Si/Al = 47. However, this is an effect of the Si/Al ratio and not of the supercell size, as is revealed by calculations with (1 × 1 × 2) supercell and Si/Al = 23. The lower Si/Al ratio leads to Al–(O–Si)_2_–O–Al sequences which are different from the isolated Al site,^60^ but the (1 × 1 × 2) supercell with Si/Al = 47 removes that sequence and only the “isolated Al site” remains. This is supported by the finding that the spectrum from the 2 × 1 × 1 supercell with Si/Al = 47 agrees with the result with Al/Si = 23. Thus, it is essential to maintain the Si/Al ratio similar to the experimental one. The effect of the supercell on the results obtained with the simple MOR(23)|bgc|0w model is also shown in Fig. 4c and it is evident that such a model is problematic, since the relative order of chemical shifts for individual Al positions depends on the choice of the supercell. We conclude that the pure supercell effect is relatively small, changing chemical shifts by less than 2 ppm in both MOR (Fig. 4c) and CHA (Fig. S12†). However, other effects confound this: (i) the Si/Al ratio, (ii) the cell symmetry, and (iii) the length of the Al–(O–Si)_*n*_–O–Al sequence. At high Si/Al ratios, Al becomes isolated^60^ and the symmetry and Al pairing effects will become negligible. However, the aluminum content at which this point is reached will depend on the framework topology.

### Cation solvation

Hydration leads to solvation of the charge-compensating cations, increasing the symmetry around the Lewis acid sites,^61^ which leads to a decrease of the electric field gradient at Al atoms. For example, the value of *C*_q_ for MOR(23)|Na|6w is up to 4 MHz less than for MOR(23)|Na|0w cases (Table S2†), and there is a 2 MHz decrease of *C*_q_ between CHA(11)|Na|0w and CHA(11)|Na|5w models. Furthermore, the high value of *C*_q_ in protonic systems, which leads in practice to the phenomenon of invisible Al, is reduced upon solvation-induced symmetrisation, causing the signal to re-appear. This phenomenon has been proven in our calculations and prior works.^62^ The results for CHA (Table S3†) and MOR (Table S2†) show that *operando* modelling with water and considering the dynamic effect improve the accuracy of the *C*_q_ prediction.

In general, the effect of the hydrated counter-cation on the local geometry of the AlO_4_ tetrahedron in hydrated silicon-rich zeolites, and thus on their ^27^Al isotropic chemical shift is negligible, as shown in previous studies with static calculations.^19,63^ Very similar chemical shifts were experimentally found for ZSM-5 exchanged with different cations (Na, Li and H)^19^ and it was concluded that in hydrated zeolites all cations are solvated, minimizing their effect on framework Al. This is commonly used in the literature as a justification of the use of background charge models in computational studies of Al NMR.^16,30,31,64^ For example, Dědeček *et al.* explained that the isotropic chemical shielding depends primarily on the local structure around Al and so the solvent and counterions do not affect the shielding by “through space” effects under hydrated conditions.^19^ However, this is only true if the cations can be considered to be fully solvated. This condition is not always met, even in hydrated samples, as the level of solvation is related to both the topology of the structure and the Si/Al ratio. We have observed in previous works that full solvent loading, considered to be approximately 1 g cm^−3^ water in the zeolite micropores, is insufficient to solvate even a single proton in cases where the pore volume is sufficiently small.^4^ Furthermore, the increasing concentration of cations with decreasing Si/Al results in fewer water molecules per cation and, consequently, only partially solvated structures appear.^65^ Such partially solvated cations affect the chemical shift by changing the local structure of the framework around Al.

Finally, different cation distributions can co-exist around a single Al site, with similar energies and hindered inter-conversion between minima, leading to multiple peaks for the same Al, which we term δ-multiplets. For example, in T1 and T2 in MOR, both sites show two different configurations with similar energy (Fig. S5†) but the cations can be solvated to different levels, due to the local environment around the cation. This is also proven by the work of Maurin *et al.*^66^ Both of these sites suffer from the presence of δ-doublets, which may be visible on the NMR spectrum and complicate the assignment of peaks to individual Al atoms.

## Conclusions

Our work shows that *operando* modeling of ^27^Al NMR spectra of zeolites, taking into account Si/Al, cation type and hydration degree, is required in order to obtain qualitatively and quantitatively correct results for complex zeolites, such as MOR. It has been shown here that testing model performance on simple zeolites such as CHA is not sufficient and may lead to qualitatively wrong conclusions, particularly when the framework is relatively symmetric and stiff and extra-framework cation positions are well defined, *e.g.*, zeolites with a *d6r* unit. First, for rigid frameworks it does not matter how the geometrical parameters are modeled, no appreciable changes in NMR are seen and, second, cations such as Na^+^ preferably occupy positions in the six-ring, so that cation solvation occurs only when a relatively large amount of water is available. However, the results presented above show that it is in general, relatively straightforward to model the spectra for a particular Al distribution in the zeolite, provided the appropriate model and method is selected: Al distribution determines the cation coordination to the framework (either metal cation or proton) and the amount of water in the sample determines the degree of solvation of the cation. NMR tensors can then be obtained as an average taken over the course of an MD simulation (Fig. 1).

Fortunately, calculations of ^27^Al chemical shifts along the MD trajectory can be avoided and replaced by a simple two-parameter correlation. Thus, the major computational bottleneck is the *ab initio* MD run, which is currently feasible for tens of ps only. If longer runs or if too many Al configurations are required for a particular study, MD simulations can be possibly performed with reliable machine learning potentials (MLPs), as recently demonstrated, *e.g.*, for siliceous and aluminosilicate zeolites.^65,67,68^ This will alleviate the sampling problem which currently hinders dynamical simulations, by extending the available timescales by several orders of magnitude. Nevertheless, the training and testing of MLPs is not straightforward and issues of transferability of MLPs between zeolites of various compositions, topologies, counterions and water content remain an active area of development.^65^

It is likely that conclusions drawn here regarding the necessity of *operando* modelling for ^27^Al NMR spectra can be generalized also for other zeolite extra-framework and framework elements (Si and O in particular) and even for different solids. The importance of *operando* modeling for zeolites is enhanced by the fact that zeolites are microporous materials and they contain water in their channels. Channel topology offers a variety of sites and coordination environments for extra-framework cations and, in addition, the solubility of individual cations also depends on the zeolite topology. It is therefore difficult to make any predictions without explicitly accounting for system-specific composition and temperature effects.

In case of zeolites with relatively low number of possible framework Al configurations, the experimental spectra can be (in principle) assigned based on the match with the simulated spectra, *e.g.*, in case of high-silica zeolites. With increasing amount of framework Al it becomes more difficult since larger number of possible configurations can occur, *e.g.*, various types of Al pairs. This situation is also obscured by the fact that with increasing amount of charge-compensating cations the water available in the channel system will not be able to solvate all cations. It remains to be seen for which samples it is possible to identify the configuration of framework aluminum from ^27^Al NMR alone. More work and collaboration between experiment and theory is required in this respect.

## Data availability

Detailed description of the regression scheme, tables of NMR calculated data, structure models, NMR referencing scheme, DFT local minima and energetics, MOR(*x*) ^27^Al NMR data (*x* = 23, 11, 7) are included in the ESI.† Datasets for NMR simulation (MD trajectories and NMR calculation input files) can be found in the zenodo database with https://doi.org/10.5281/zenodo.7708843.

## Author contributions

The manuscript was written through contributions of all authors. All the authors have given approval to the final version of the manuscript.

## Conflicts of interest

There are no conflicts to declare.

## Supplementary Material

SC-014-D3SC02492J-s001

## References

[cit1] Grajciar L., Heard C. J., Bondarenko A. A., Polynski M. V., Meeprasert J., Pidko E. A., Nachtigall P. (2018). Towards operando computational modeling in heterogeneous catalysis. Chem. Soc. Rev..

[cit2] Nimlos C. T., Hoffman A. J., Hur Y. G., Lee B. J., Di Iorio J. R., Hibbitts D. D., Gounder R. (2020). Experimental and Theoretical Assessments of Aluminum Proximity in MFI Zeolites and Its Alteration by Organic and Inorganic Structure-Directing Agents. Chem. Mater..

[cit3] Knott B. C., Nimlos C. T., Robichaud D. J., Nimlos M. R., Kim S., Gounder R. (2018). Consideration of the Aluminum Distribution in Zeolites in Theoretical and Experimental Catalysis Research. ACS Catal..

[cit4] Heard C. J., Grajciar L., Nachtigall P. (2019). The effect of water on the validity of Löwenstein's rule. Chem. Sci..

[cit5] Heard C. J., Grajciar L., Rice C. M., Pugh S. M., Nachtigall P., Ashbrook S. E., Morriss R. E. (2019). Fast room temperature lability of aluminosilicate zeolites. Nat. Commun..

[cit6] Fernandez C. and Pruski M., Solid State NMR, ed. J. C. C. Chan, Springer Berlin Heidelberg, Berlin, Heidelberg, 2012, pp. 119–188, 10.1007/128_2011_141

[cit7] Ashbrook S. E., Griffin J. M., Johnston K. E. (2018). Recent Advances in Solid-State Nuclear Magnetic Resonance Spectroscopy. Annu. Rev. Anal. Chem..

[cit8] Xin S., Wang Q., Xu J., Chu Y., Wang P., Feng N., Qi G., Trebosc J., Lafon O., Fan W., Deng F. (2019). The acidic nature of “NMR-invisible” tri-coordinated framework aluminum species in zeolites. Chem. Sci..

[cit9] Dib E., Mineva T., Veron E., Sarou-Kanian V., Fayon F., Alonso B. (2018). ZSM-5 Zeolite: Complete Al Bond Connectivity and Implications on Structure Formation from Solid-State NMR and Quantum Chemistry Calculations. J. Phys. Chem. Lett..

[cit10] Al-Nahari S., Dib E., Cammarano C., Saint-Germes E., Massiot D., Sarou-Kanian V., Alonso B. (2023). Impact of Mineralizing Agents on Aluminum Distribution and Acidity of ZSM-5 Zeolites. Angew. Chem., Int. Ed..

[cit11] Wang W., Xu J., Deng F. (2022). Recent advances in solid-state NMR of zeolite catalysts. Natl. Sci. Rev..

[cit12] Yakimov A. V., Ravi M., Verel R., Sushkevich V. L., van Bokhoven J. A., Copéret C. (2022). Structure and Framework Association of Lewis Acid Sites in MOR Zeolite. J. Am. Chem. Soc..

[cit13] Ravi M., Sushkevich V. L., van Bokhoven J. A. (2019). Lewis Acidity Inherent to the Framework of Zeolite Mordenite. J. Phys. Chem. C.

[cit14] Ravi M., Sushkevich V. L., van Bokhoven J. A. (2021). On the location of Lewis acidic aluminum in zeolite mordenite and the role of framework-associated aluminum in mediating the switch between Brønsted and Lewis acidity. Chem. Sci..

[cit15] Fan B., Zhu D., Wang L., Xu S., Wei Y., Liu Z. (2022). Dynamic evolution of Al species in the hydrothermal dealumination process of CHA zeolites. Inorg. Chem. Front..

[cit16] Sklenak S., Dědeček J., Li C., Wichterlová B., Gábová V., Sierka M., Sauer J. (2007). Aluminum siting in silicon-rich zeolite frameworks: a combined high-resolution ^27^Al NMR spectroscopy and quantum mechanics/molecular mechanics study of ZSM-5. Angew. Chem., Int. Ed. Engl..

[cit17] Chen K., Gan Z., Horstmeier S., White J. L. (2021). Distribution of Aluminum Species in Zeolite Catalysts: ^27^Al NMR of Framework, Partially-Coordinated Framework, and Non-Framework Moieties. J. Am. Chem. Soc..

[cit18] Chen K., Horstmeier S., Nguyen V. T., Wang B., Crossley S. P., Pham T., Gan Z., Hung I., White J. L. (2020). Structure and Catalytic Characterization of a Second Framework Al(IV) Site in Zeolite Catalysts Revealed by NMR at 35.2 T. J. Am. Chem. Soc..

[cit19] Sklenak S., Dědeček J., Li C., Wichterlová B., Gábová V., Sierka M., Sauer J. (2009). Aluminium siting in the ZSM-5 framework by combination of high resolution ^27^Al NMR and DFT/MM calculations. Phys. Chem. Chem. Phys..

[cit20] Kučera J., Nachtigall P. (2005). A simple correlation between average T–O–T angles and ^27^Al NMR chemical shifts does not hold in high-silica zeolites. Microporous Mesoporous Mater..

[cit21] Kučera J., Nachtigall P. (2005). ^27^Al NMR chemical shifts do not correlate with average T-O-T angles: Theoretical study of MCM-58 zeolite. Stud. Surf. Sci. Catal..

[cit22] Mazurek A. H., Szeleszczuk Ł., Pisklak D. M. (2021). A Review on Combination of *Ab Initio* Molecular Dynamics and NMR Parameters Calculations. Int. J. Mol. Sci..

[cit23] Blanc F., Middlemiss D. S., Buannic L., Palumbo J. L., Farnan I., Grey C. P. (2012). Thermal phase transformations in LaGaO_3_ and LaAlO_3_ perovskites: An experimental and computational solid-state NMR study. Solid State Nucl. Magn. Reson..

[cit24] Dračínský M., Bouř P., Hodgkinson P. (2016). Temperature Dependence of NMR Parameters Calculated from Path Integral Molecular Dynamics Simulations. J. Chem. Theory Comput..

[cit25] Dračínský M., Hodgkinson P. (2013). A molecular dynamics study of the effects of fast molecular motions on solid-state NMR parameters. CrystEngComm.

[cit26] Dračínský M., Bouř P. (2012). Vibrational averaging of the chemical shift in crystalline α-glycine. J. Comput. Chem..

[cit27] Folliet N., Roiland C., Bégu S., Aubert A., Mineva T., Goursot A., Selvaraj K., Duma L., Tielens F., Mauri F., Laurent G., Bonhomme C., Gervais C., Babonneau F., Azaïs T. (2011). Investigation of the Interface in Silica-Encapsulated Liposomes by Combining Solid State NMR and First Principles Calculations. J. Am. Chem. Soc..

[cit28] Vanlommel S., Hoffman A. E. J., Smet S., Radhakrishnan S., Asselman K., Chandran C. V., Breynaert E., Kirschhock C. E. A., Martens J. A., Van Speybroeck V. (2022). How Water and Ion Mobility Affect the NMR Fingerprints of the Hydrated JBW Zeolite: A Combined Computational-Experimental Investigation. Chem.–Eur. J..

[cit29] Mlekodaj K., Dedecek J., Pashkova V., Tabor E., Klein P., Urbanova M., Karcz R., Sazama P., Whittleton S. R., Thomas H. M., Fishchuk A. V., Sklenak S. (2019). Al Organization in the SSZ-13 Zeolite. Al Distribution and Extraframework Sites of Divalent Cations. J. Phys. Chem. C.

[cit30] Göltl F., Love A. M., Schuenzel S. C., Wolf P., Mavrikakis M., Hermans I. (2019). Computational description of key spectroscopic features of zeolite SSZ-13. Phys. Chem. Chem. Phys..

[cit31] Holzinger J., Nielsen M., Beato P., Brogaard R. Y., Buono C., Dyballa M., Falsig H., Skibsted J., Svelle S. (2019). Identification of Distinct Framework Aluminum Sites in Zeolite ZSM-23: A Combined Computational and Experimental ^27^Al NMR Study. J. Phys. Chem. C.

[cit32] Kresse G., Hafner J. (1993). Ab initio molecular dynamics for open-shell transition metals. Phys. Rev. B: Condens. Matter Mater. Phys..

[cit33] Kresse G., Furthmuller J. (1996). Efficiency of *ab initio* total energy calculations for metals and semiconductors using a plane-wave basis set. Comput. Mater. Sci..

[cit34] Kresse G., Furthmuller J. (1996). Efficient iterative schemes for *ab initio* total-energy calculations using a plane-wave basis set. Phys. Rev. B: Condens. Matter Mater. Phys..

[cit35] Kresse G., Joubert D. (1999). From ultrasoft pseudopotentials to the projector augmented-wave method. Phys. Rev. B: Condens. Matter Mater. Phys..

[cit36] Perdew J. P., Burke K., Ernzerhof M. (1996). Generalized Gradient Approximation Made Simple. Phys. Rev. Lett..

[cit37] Grimme S., Ehrlich S., Goerigk L. (2011). Effect of the damping function in dispersion corrected density functional theory. J. Comput. Chem..

[cit38] Gillan M. J., Alfe D., Michaelides A. (2016). Perspective: How good is DFT for water?. J. Chem. Phys..

[cit39] Pickard C. J., Mauri F. (2001). All-electron magnetic response with pseudopotentials: NMR chemical shifts. Phys. Rev. B: Condens. Matter Mater. Phys..

[cit40] Yates J. R., Pickard C. J., Mauri F. (2007). Calculation of NMR chemical shifts for extended systems using ultrasoft pseudopotentials. Phys. Rev. B: Condens. Matter Mater. Phys..

[cit41] Csonka G. I., Perdew J. P., Ruzsinszky A., Philipsen P. H. T., Lebègue S., Paier J., Vydrov O. A., Ángyán J. G. (2009). Assessing the performance of recent density functionals for bulk solids. Phys. Rev. B: Condens. Matter Mater. Phys..

[cit42] Hartman J. D., Kudla R. A., Day G. M., Mueller L. J., Beran G. J. (2016). Benchmark fragment-based ^1^H, ^13^C, ^15^N and ^17^O chemical shift predictions in molecular crystals. Phys. Chem. Chem. Phys..

[cit43] Sun H., Dwaraknath S., Ling H., Qu X., Huck P., Persson K. A., Hayes S. E. (2020). Enabling materials informatics for ^29^Si solid-state NMR of crystalline materials. npj Comput. Mater..

[cit44] Hjorth Larsen A., Jorgen Mortensen J., Blomqvist J., Castelli I. E., Christensen R., Dulak M., Friis J., Groves M. N., Hammer B., Hargus C., Hermes E. D., Jennings P. C., Bjerre Jensen P., Kermode J., Kitchin J. R., Leonhard Kolsbjerg E., Kubal J., Kaasbjerg K., Lysgaard S., Bergmann Maronsson J., Maxson T., Olsen T., Pastewka L., Peterson A., Rostgaard C., Schiotz J., Schutt O., Strange M., Thygesen K. S., Vegge T., Vilhelmsen L., Walter M., Zeng Z., Jacobsen K. W. (2017). The atomic simulation environment-a Python library for working with atoms. J. Phys.: Condens. Matter.

[cit45] Pedregosa F., Varoquaux G., Gramfort A., Michel V., Thirion B., Grisel O., Blondel M., Prettenhofer P., Weiss R., Dubourg V., Vanderplas J., Passos A., Cournapeau D., Brucher M., Perrot M., Duchesnay E. (2011). Scikit-learn: Machine Learning in Python. J. Mach. Learn. Res..

[cit46] Grey C. P., Vega A. J. (1995). Determination of the Quadrupole Coupling Constant of the Invisible Aluminum Spins in Zeolite HY with ^1^H/^27^Al TRAPDOR NMR. J. Am. Chem. Soc..

[cit47] Kentgens A. P. M., Iuga D., Kalwei M., Koller H. (2001). Direct Observation of Brønsted Acidic Sites in Dehydrated Zeolite H-ZSM5 Using DFS-Enhanced ^27^Al MQMAS NMR Spectroscopy. J. Am. Chem. Soc..

[cit48] Ernst H., Freude D., Wolf I. (1993). Multinuclear Solid-State NMR Studies of Brønsted Sites in Zeolites. Chem. Phys. Lett..

[cit49] Klein P., Pashkova V., Thomas H. M., Whittleton S. R., Brus J., Kobera L., Dedecek J., Sklenak S. (2016). Local Structure of Cationic Sites in Dehydrated Zeolites Inferred from ^27^Al Magic-Angle Spinning NMR and Density Functional Theory Calculations. A Study on Li-, Na-, and K-Chabazite. J. Phys. Chem. C.

[cit50] Lippmaa E., Samoson A., Magi M. (1986). High-resolution ^27^Al NMR of aluminosilicates. J. Am. Chem. Soc..

[cit51] Li Y., Yu J., Xu R. (2013). Criteria for Zeolite Frameworks Realizable for Target Synthesis. Angew. Chem., Int. Ed..

[cit52] Huntley G. M., Luck R. L., Mullins M. E., Newberry N. K. (2021). Hydrochloric Acid Modification and Lead Removal Studies on Naturally Occurring Zeolites from Nevada, New Mexico, and Arizona. Processes.

[cit53] Holzinger J., Beato P., Lundegaard L. F., Skibsted J. (2018). Distribution of Aluminum over the Tetrahedral Sites in ZSM-5 Zeolites and Their Evolution after Steam Treatment. J. Phys. Chem. C.

[cit54] Willimetz D. , Theoretical investigation of ^27^Al chemical shifts dependence on water amount and temperature in zeolite MFI, Bachelor thesis, Charles University, https://dspace.cuni.cz/bitstream/handle/20.500.11956/181975/130361065.pdf?sequence=1&isAllowed=y, 2023

[cit55] Gaumard R., Dragún D., Pedroza-Montero J. N., Alonso B., Guesmi H., Malkin Ondík I., Mineva T. (2022). Regression Machine Learning Models Used to Predict DFT-Computed NMR Parameters of Zeolites. Computation.

[cit56] Venetos M. C., Wen M., Persson K. A. (2023). Machine Learning Full NMR Chemical Shift Tensors of Silicon Oxides with Equivariant Graph Neural Networks. J. Phys. Chem. A.

[cit57] Cuny J., Xie Y., Pickard C. J., Hassanali A. A. (2016). Ab Initio Quality NMR Parameters in Solid-State Materials Using a High-Dimensional Neural-Network Representation. J. Chem. Theory Comput..

[cit58] Schroeder C., Siozios V., Mück-Lichtenfeld C., Hunger M., Hansen M. R., Koller H. (2020). Hydrogen Bond Formation of Bronsted Acid Sites in Zeolites. Chem. Mater..

[cit59] Hack J. H., Dombrowski J. P., Ma X., Chen Y., Lewis N. H. C., Carpenter W. B., Li C., Voth G. A., Kung H. H., Tokmakoff A. (2021). Structural Characterization of Protonated Water Clusters Confined in HZSM-5 Zeolites. J. Am. Chem. Soc..

[cit60] Dědeček J., Sobalík Z., Wichterlová B. (2012). Siting and Distribution of Framework Aluminium Atoms in Silicon-Rich Zeolites and Impact on Catalysis. Catal. Rev.: Sci. Eng..

[cit61] Zhu L., Seff K., Olson D. H., Cohen B. J., Von Dreele R. B. (1999). Hydronium Ions in Zeolites. 1. Structures of Partially and Fully Dehydrated Na,H_3_O−X by X-ray and Neutron Diffraction. J. Phys. Chem. B.

[cit62] Vjunov A., Wang M., Govind N., Huthwelker T., Shi H., Mei D., Fulton J. L., Lercher J. A. (2017). Tracking the Chemical Transformations at the Brønsted Acid Site upon Water-Induced Deprotonation in a Zeolite Pore. Chem. Mater..

[cit63] Sarv P., Fernandez C., Amoureux J.-P., Keskinen K. (1996). Distribution of Tetrahedral Aluminium Sites in ZSM-5 Type Zeolites: An ^27^Al (Multiquantum) Magic Angle Spinning NMR Study. J. Phys. Chem..

[cit64] Dedecek J., Lucero M. J., Li C. B., Gao F., Klein P., Urbanova M., Tvaruzkova Z., Sazama P., Sklenak S. (2011). Complex Analysis of the Aluminum Siting in the Framework of Silicon-Rich Zeolites. A Case Study on Ferrierite. J. Phys. Chem. C.

[cit65] Saha I. E. , Erlebach A., Nachtigall P., Heard C. J. and Grajciar L., Reactive Neural Network Potential for Aluminosilicate Zeolites and Water: Quantifying the Effect of Si/Al Ratio on Proton Solvation and Water Diffusion in H-FAU, ChemRxiv, 2022, 10.26434/chemrxiv-2022-d1sj9

[cit66] Maurin G., Bell R. G., Devautour S., Henn F., Giuntini J. C. (2004). Modeling the Effect of Hydration in Zeolite Na^+^−Mordenite. J. Phys. Chem. B.

[cit67] Erlebach A., Nachtigall P., Grajciar L. (2022). Accurate large-scale simulations of siliceous zeolites by neural network potentials. npj Comput. Mater..

[cit68] Erlebach A. , Šípka M., Saha I., Nachtigall P., Heard C. J. and Grajciar L., A reactive neural network framework for water-loaded acidic zeolites, arXiv, 2023, preprint, arXiv:2307.00911, 10.48550/arXiv.2307.00911 PMC11101627 38760371

