# Correction: The need for *operando* modelling of ^27^Al NMR in zeolites: the effect of temperature, topology and water†

**DOI:** 10.1039/d3sc90195e

**Published:** 2023-11-22

**Authors:** Chen Lei, Andreas Erlebach, Federico Brivio, Lukáš Grajciar, Zdeněk Tošner, Christopher J. Heard, Petr Nachtigall

**Affiliations:** a Department of Physical and Macromolecular Chemistry, Faculty of Science, Charles University in Prague 128 43 Prague 2 Czech Republic heardc@natur.cuni.cz

## Abstract

Correction for ‘The need for *operando* modelling of ^27^Al NMR in zeolites: the effect of temperature, topology and water’ by Chen Lei *et al.*, *Chem. Sci.*, 2023, **14**, 9101–9113, https://doi.org/10.1039/D3SC02492J.

The authors regret that the Acknowledgements section of the original article was incomplete. The complete acknowledgements are shown in the following text:

Charles University Centre of Advanced Materials (CUCAM) (OP VVV Excellent Research Teams, project number CZ.02.1.01/0.0/0.0/15_003/0000417) is acknowledged. This work was supported by the Ministry of Education, Youth and Sports of the Czech Republic through the e-INFRA CZ (ID: 90254). The authors acknowledge the Czech Science Foundation through projects 20-12099S (C. L, Z. T, P. N) and 23-07616S (CJH, LG). CJH and LG acknowledge support from the Charles University Centre of Excellence award UNCE/SCI/014. The authors acknowledge Daniel Willimetz for additional generalization tests and the verification of ML methods.

The Royal Society of Chemistry apologises for these errors and any consequent inconvenience to authors and readers.

## Supplementary Material

