# Supplementary material for: The need for operando modelling of 27Al NMR in zeolites: the effect of temperature, topology and water
Source: Chem Sci. 2023 Aug 3;14(34):9101–13. doi: 10.1039/d3sc02492j (PMC10466278; doi:10.1039/d3sc02492j)
Supplement: SC-014-D3SC02492J-s001 [file SC-014-D3SC02492J-s001.pdf]

Electronic Supplementary Material (ESI) for Chemical Science.
This journal is © The Royal Society of Chemistry 2023

Supporting Information

The need for operando modelling of ^{27}Al NMR in zeolites: The effect of Temperature, Topology and Water

Chen Lei, Andreas Erlebach, Federico Brivio, Lukáš Grajciar, Zdeněk Tošner, Christopher J. Heard, Petr Nachtigall†*

Department of Physical and Macromolecular Chemistry, Charles University, Hlavova 8, Prague 2, Prague 128 43, Czech Republic

*E-mail: heardc@natur.cuni.cz

†deceased, 28th December 2022

Models

To balance the negative charge of the framework due to introducing the Al substitution, extra framework cations are needed, and there are three options for computation here: 1) background charge (bgc), 2) alkali metal cations (Na is used here), 3) proton (H). The notation of the models, ABC(n)|M|xw, denotes the zeolite type ABC with Si/Al ratio n and x water molecules in each cell. The range of the maximum content of water within the confined microspore volumes of zeolites corresponds to the concentration of 1 g cm^{-1}, five water molecules per cell in CHA primitive cell and six in MOR. It is known that zeolites are very easy to hydrate, therefore the case with one water molecule in the CHA single cell, CHA(11)|M|1w, is also used for dehydrated models. The result of CHA(11)|Na|1w does not show much difference from the CHA(11)|Na|0w case. Although the CHA(11)|H|1w shows differences from the CHA(11)|H|0w because of the hydrogen bonding interaction between the water and the framework(Table S6), the quadrupole coupling is still too large to observe the peaks. Therefore dehydrated H-MOR is not considered.

^{27}Al NMR LASSO regression in zeolites

The least absolute shrinkage and selection operator (LASSO) regression was applied to select the most relevant structural features for linear correlation of DFT calculated ^{27}Al chemical shielding σ and the atomic environments of the Al atoms. LASSO regression minimizes an objective function L with the error sum of squares over n samples between target values $\sigma_{i,DFT}$ and a linear model $\sum_{j=1}^{p} c_j \cdot x_{ij}$ with p coefficients c_j and the structural features x_{ij}:

$$L = \frac{1}{n}\sum_{i=1}^{n}\left(\sigma_{i,DFT} - \sum_{j=1}^{p} c_j \cdot x_{ij}\right)^2 + \lambda\left(\sum_{j=1}^{p} |c_j|\right). \quad (1)$$

The L1 regularization term $\lambda\left(\sum_{j=1}^{p} |c_j|\right)$ with hyperparameter λ acts as penalization of c_j allowing them to go to zero during minimization of L and, therefore, feature selection. All LASSO regressions used regularization parameters $\lambda \in [10^0, 10^{-3}]$ and 80% of DFT calculations for training and the remaining data points as test set.

Table S1. Local (framework) features considered in LASSO regression including average distances d and angles α as well as the corresponding difference (Δd, $\Delta\alpha$) between minimum and maximum

values (spread) of the features in the $(AlO_{4/2})^-$ tetrahedra. Additionally, four average distances to extra-framework atoms were considered.

Framework features

$d_{\text{Al-O}}$	Mean Al-O distance
$d_{\text{Al-Si}}$	Mean Al-Si distance
$\alpha_{\text{O-Al-O}}$	Mean O-Al-O angle
$\alpha_{\text{Al-O-Si}}$	Mean Al-O-Si angle
$\Delta d_{\text{Al-O}}$	Spread of Al-O distances
$\Delta d_{\text{Al-Si}}$	Spread of Al-Si distances
$\Delta\alpha_{\text{O-Al-O}}$	Spread of O-Al-O angles
$\Delta\alpha_{\text{Al-O-Si}}$	Spread of Al-O-Si angles

Extra-framework features

$d_{\text{Na-O}}$	Mean Na-O distance (O neighboring Al)
$d_{\text{Al-Na}}$	Mean Al-Na distance
$d_{\text{Na-Ow}}$	Mean Na-O distance (water O neighboring Na)
$d_{\text{Al-Ow}}$	Mean Al-O distance (water O)

Initially, LASSO regression used twelve structural features (see Table S1) and about 700 DFT NMR calculations of ⟨CHA(11)|Na|5W⟩ to test the importance of local (framework) descriptors of $(AlO_{4/2})^-$ tetrahedra and extra-framework descriptors such as Na-Al distances. It turned out that seven framework features (all but $\Delta\alpha_{\text{O-Al-O}}$) yield a coefficient of determination R^2 of 0.85 and using additional descriptors provide virtually no improvement (see Fig. S1). Next, a second LASSO regression was used to find the most relevant framework features employing a larger dataset (1466 DFT calculations) also including dehydrated and H-form CHA and MOR (⟨CHA(11)|Na|0w⟩, ⟨CHA(11)|Na|5w⟩, ⟨CHA(11)|H|5w⟩, ⟨CHA(11)|H|5w⟩, ⟨CHA(3)|H|0w⟩, ⟨CHA(3)|H|6w⟩, ⟨MOR(11)|Na|1w⟩, ⟨MOR(11)|Na|5w⟩). The two leading structural features $d_{\text{Al-O}}$ and $\alpha_{\text{Al-O-Si}}$ provided sufficient accuracy (see Fig. S9) and were used for (unregularized) linear regression. The resulting linear model:

$$\sigma = c_1 d_{Al-O} + c_2 \alpha_{Al-O-Si} + c_3, \qquad (2)$$

with coefficients c_1 = 180.5986 ppm/Å; c_2 = 0.7997 ppm/deg; c_3 = 64.1503 ppm is depicted in Fig. S10a and shows an R^2 of 0.89 and an RMSE of 1.7 ppm (see Fig. **S**10b). In addition, we fitted a

linear model (see Fig. S8c and d) only depending on $\alpha_{Al-O-Si}$ as proposed by Lippmaa (σ =0.6662$\alpha_{Al\text{-}O\text{-}Si}$ + 399.5362) resulting in considerably higher RMSE of 4.2 ppm (R^2 = 0.36).

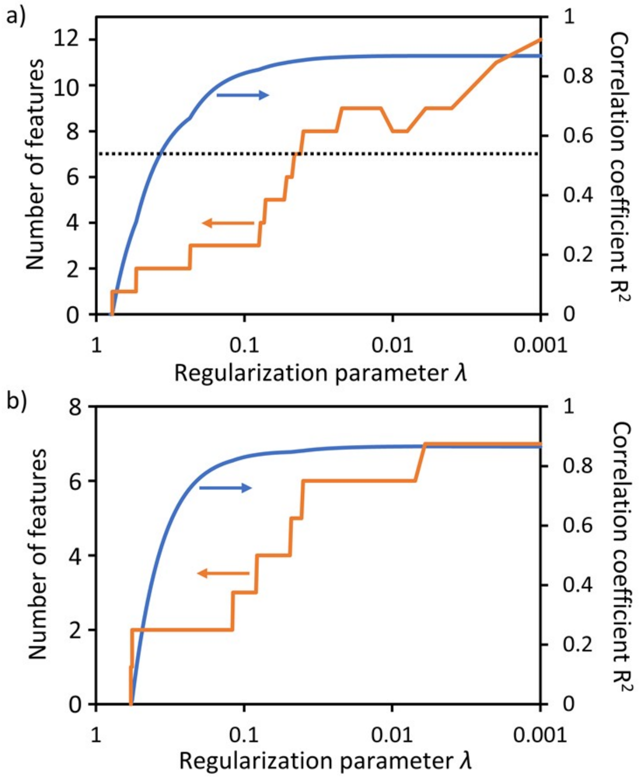

Fig. S1 Coefficient of determination R^2 of the employed test set and the number of structural features with non-zero coefficients as a function of the LASSO regularization parameter λ for (a) the initial LASSO regression using ⟨CHA(11)|Na|5W⟩ and all 12 structural features (see Table S1) and (b) LASSO regression using (hydrated and dehydrated) CHA and MOR data in H and Na-form.

Table S2. ^{27}Al NMR parameters of MOR. The model for MOR with Si/Al=11 has Al in T1 and T3 sites. The model for MOR with Si/Al=7 has Al in T1, T3 and T4 sites.

Models	δ (ppm)				C_q (MHz)				<δ> (ppm)			
	T1	T2	T3	T4	T1	T2	T3	T4	T1	T2	T3	T4
MOR(47)|bcg|0w	52.6	50.2	51.8	54.1	3.26	2.72	2.43	3.05				
MOR(23)|bcg|0w	49.7	49.5	52.1	56.0	2.71	2.71	2.82	3.33				
MOR(23)|Na|0w	53.1	53.6	54.9	59.8	5.86	6.72	5.41	5.60			55.3	
	59.9^a	53.7^a			5.65^a	6.29^a			59.3			
MOR(23)|Na|6w	52.2	51.6	56.1	61.7	4.98	2.56	3.86	7.71	54.6	54.3	56.0	58.9
MOR(23)|Na|6w*	50.7	52.4	50.4	55.9	3.78	2.24	4.74	2.15	54.5	53.3	52.2	57.0
MOR(11)|Na|5w	52.9		56.2		4.62		5.17		54.3		56.1	
MOR(7)|Na|1w	53.8		57.7	61.4	4.20		5.16	6.99	53.2		56.2	61.5
MOR(7)|Na|5w	51.8		55.8	60.3	4.24		4.57	3.23	54.0		56.2	60.0
MOR(7)|H|5w^b	55.8		50.2	57.7	13.29		8.91	3.24				
MOR(7)|H|5w*^{b}	52.6		55.1	59.0	5.97		4.23	6.97	54.6		53.6	58.1

Note: MOR(23)|Na|6w and MOR(23)|Na|6w* denote the states of the Na^+ are partial solvated and completely-solvated as shown in Fig. 2; C_q are from the static calculation. <δ> refers to the chemical shift averaged over the MD trajectory. C_q is the quadrupolar coupling constant from static calculations. a: two nearly isoenergetic configurations for Na were found for Al at T1 in MOR(23)|Na|0w and Al at T2 in MOR(23)|Na|0w. Static chemical shifts and C_q values were calculated for both configurations. For dehydrated Na-MOR, dynamic simulations were not run, as tests showed the effect of dynamics was small, due to the lack of Na motion during the simulations. b: in MOR(7)|H|5w, all three protons originating at T1, T3 and T4 are preferentially solvated in the static model(MOR(7)|H|5w*), but there is a competing configuration at +7 kJ mol^{-1} (MOR(7)|H|5w), in which T1 and T3 protons are unsolvated. The protons at T1 and T3 rapidly fluctuate between solvated and unsolvated configurations along the MD trajectory.

Table S3. Calculated ^{27}Al NMR parameters of CHA (Si/Al=11) with a fixed volume of 796.35 Å^3, which is the optimal volume for the Na|5w model.

CHA	σ (ppm)	δ (ppm)	C_q(MHz)	$\langle\sigma\rangle$ (ppm)	$\langle\delta\rangle$ (ppm)
bgc|0w	494.20	61.2	2.75	-	-
Na|0w	494.39	61.0	6.70	493.25	62.2
Na|5w	493.44	62.0	5.31	492.42	63.1
Na|5w*	494.85	60.5	3.19	493.81	61.5
H|0w	498.06	56.9	19.51	496.87	58.2
H|5w	494.04	61.3	1.44	493.59	61.8

*The case with completely-solvated Na^+ case. Its energy is only **9.6** kJ/mol lower than the Na|5w case with partially solvated Na^+; the $\langle\sigma\rangle$ is obtained from the trajectory with fully solvated (as shown in Fig. S9). The CHA|H|0w model used here has H at the O4 BAS site, defined according to the convention in reference[1].

Table S4. The geometric descriptors and their contributions to the chemical shielding for the CHA|Na|0w model with varying volumes. The definitions of all the descriptors are given in Table S1.

	Geometrical descriptors					Contribution to the ^{27}Al σ (ppm)		^{27}Al δ (ppm)	
V(Å3)	d_{Al-O} (Å)	Δd_{Al-O} (Å)	d_{Al-Si} (Å)	$\alpha_{Al-0-Si}$ (°)	α_{O-Al-O} (°)	$\sigma(d_{Al-O})$	$\sigma(\alpha_{Al-O-Si})$	δ(FIT)	δ(DFT)
810.90	1.742	0.069	3.706	143.26	109.40	314.7	114.6	62.0	60.8
806.04	1.741	0.070	3.699	142.97	109.39	314.5	114.3	62.5	61.6
801.20	1.741	0.068	3.695	142.71	109.38	314.3	114.1	62.9	62.2
796.39	1.740	0.066	3.689	142.47	109.37	314.2	113.9	63.3	62.8
791.59	1.739	0.064	3.683	142.28	109.36	314.0	113.8	63.6	63.4
786.81	1.738	0.062	3.678	142.11	109.36	313.9	113.6	63.9	63.8
763.21	1.736	0.046	3.652	141.28	109.35	313.5	113.0	65.1	65.7

Table S5. The geometric descriptors and their contribution to the NMR chemical shielding of CHA with the fixed volume (796.35 Å^3) based on the LASSO analysis. Note: all the CHA|H|0w case in here are used the same model with BAS site on O4.

Models	Geometrical descriptors					^{27}Al σ (ppm)		^{27}Al δ (ppm)	
CHA(11)	$d_{Al\text{-}O}$(Å)	$\Delta d_{Al\text{-}O}$ (Å)	$d_{Al\text{-}Si}$ (Å)	$\alpha_{Al\text{-}O\text{-}Si}$ (°)	$\alpha_{O\text{-}Al\text{-}O}$ (°)	$\sigma(d_{Al\text{-}O})$	$\sigma(\alpha_{Al\text{-}O\text{-}Si})$	δ (DFT)	δ(FIT)
bgc|0w	1.742	0.028	3.677	141.78	109.472	314.5	113.4	61.1	63.5
Na|0w	1.743	0.075	3.679	141.551	109.464	314.7	113.2	60.9	63.5
<Na|0w>	1.745	0.103	3.628	141.650	109.367	315.1	113.3	62.2	62.9
Na|5w	1.745	0.051	3.676	141.526	109.470	315.1	112.3	62.0	64.0
<Na|5w>	1.752	0.070	3.623	139.493	109.385	315.9	111.7	63.1	63.9
Na|5w*	1.745	0.051	3.676	141.526	109.470	315.1	113.2	60.4	63.0
<Na|5w>*	1.752	0.090	3.623	139.493	109.385	316.4	111.6	62.4	63.4
H|0w	1.760	0.211	3.422	143.318	108.798	317.9	114.7	56.9	58.4
<H|0w>	1.765	0.238	3.551	142.402	108.729	318.8	113.9	58.2	58.2
H|5w	1.749	0.037	3.676	140.858	109.470	315.9	112.7	61.3	62.8
<H|5w>	1.751	0.086	3.625	140.232	109.383	316.2	112.2	61.8	63.0

Table S6. The geometric descriptors and their contribution to the ^{27}Al NMR σ parameters for static and average MD NMR calculation in MOR.

<table>
<tr><th></th><th>Models</th><th>$d_{Al\text{-}O}$(Å)</th><th>$\Delta d_{Al\text{-}O}$(Å)</th><th>$d_{Al\text{-}Si}$(Å)</th><th>$\alpha_{Al\text{-}O\text{-}Si}$(°)</th><th>$\alpha_{O\text{-}Al\text{-}O}$(°)</th><th>$\sigma(d_{Al\text{-}O})$ (ppm)</th><th>$\sigma(\alpha_{Al\text{-}O\text{-}Si})$ (ppm)</th><th>δ(ML) (ppm)</th><th>δ(DFT)/ ppm</th></tr>
<tr><td rowspan="9">T1</td><td>MOR(23)Na|0w</td><td>1.751</td><td>0.060</td><td>3.43</td><td>153.29</td><td>109.36</td><td>316.2</td><td>122.6</td><td>51.4</td><td>53.1</td></tr>
<tr><td>MOR(23)|Na|6w</td><td>1.751</td><td>0.048</td><td>3.42</td><td>153.17</td><td>109.39</td><td>316.2</td><td>122.5</td><td>51.5</td><td>52.2</td></tr>
<tr><td><MOR(23)|Na|6w></td><td>1.754</td><td>0.105</td><td>3.43</td><td>150.04</td><td>109.35</td><td>316.8</td><td>120.0</td><td>53.6</td><td>54.6</td></tr>
<tr><td>MOR(23)|Na|6w*</td><td>1.750</td><td>0.054</td><td>3.44</td><td>152.51</td><td>109.46</td><td>316.0</td><td>122.0</td><td>52.3</td><td>50.8</td></tr>
<tr><td><MOR(23)|Na|6w>*</td><td>1.751</td><td>0.094</td><td>3.43</td><td>149.01</td><td>109.39</td><td>316.3</td><td>119.2</td><td>55.1</td><td>54.4</td></tr>
<tr><td>MOR(23)Na|0w [(a)]</td><td>1.754</td><td>0.072</td><td>3.40</td><td>141.05</td><td>109.28</td><td>316.7</td><td>112.8</td><td>61.7</td><td>59.9</td></tr>
<tr><td>MOR(7)|H|5w</td><td>1.756</td><td>0.147</td><td>3.44</td><td>149.20</td><td>109.24</td><td>317.1</td><td>119.4</td><td>54.1</td><td>55.8</td></tr>
<tr><td>MOR(7)|H|5w*</td><td>1.752</td><td>0.074</td><td>3.44</td><td>151.41</td><td>109.39</td><td>316.4</td><td>121.1</td><td>52.9</td><td>52.6</td></tr>
<tr><td><MOR(7)|H|5w></td><td>1.760</td><td>0.152</td><td>3.45</td><td>149.16</td><td>109.23</td><td>317.8</td><td>119.3</td><td>53.3</td><td>54.6</td></tr>
<tr><td rowspan="4">T2</td><td>MOR(23)Na|0w</td><td>1.754</td><td>0.063</td><td>3.62</td><td>148.52</td><td>109.05</td><td>316.8</td><td>118.8</td><td>54.9</td><td>53.5</td></tr>
<tr><td>MOR(23)|Na|6w</td><td>1.746</td><td>0.032</td><td>3.74</td><td>152.01</td><td>109.39</td><td>315.3</td><td>121.6</td><td>53.5</td><td>51.6</td></tr>
<tr><td><MOR(23)|Na|6w></td><td>1.752</td><td>0.092</td><td>3.60</td><td>149.90</td><td>109.32</td><td>316.4</td><td>119.9</td><td>54.2</td><td>54.3</td></tr>
<tr><td><MOR(23)|Na|6w>*</td><td>1.747</td><td>0.086</td><td>3.62</td><td>151.89</td><td>109.38</td><td>315.4</td><td>121.5</td><td>53.5</td><td>53.2</td></tr>
<tr><td rowspan="4">T3</td><td>MOR(23)Na|0w</td><td>1.751</td><td>0.049</td><td>3.44</td><td>147.40</td><td>109.36</td><td>316.2</td><td>117.9</td><td>56.6</td><td>54.9</td></tr>
<tr><td>MOR(23)|Na|6w</td><td>1.750</td><td>0.042</td><td>3.62</td><td>146.65</td><td>109.43</td><td>316.1</td><td>117.3</td><td>57.4</td><td>56.1</td></tr>
<tr><td><MOR(23)|Na|6w></td><td>1.757</td><td>0.093</td><td>3.55</td><td>146.44</td><td>109.36</td><td>317.2</td><td>117.2</td><td>56.3</td><td>56.0</td></tr>
<tr><td><MOR(23)|Na|6w>*</td><td>1.752</td><td>0.085</td><td>3.52</td><td>149.49</td><td>109.38</td><td>316.4</td><td>119.6</td><td>54.5</td><td>52.2</td></tr>
</table>

<table>
<tr><td></td><td>MOR(7)|H|5w</td><td>1.753</td><td>0.111</td><td>3.60</td><td>149.89</td><td>109.28</td><td>316.6</td><td>119.9</td><td>54.0</td><td>50.2</td></tr>
<tr><td></td><td>MOR(7)|H|5w*</td><td>1.748</td><td>0.088</td><td>3.39</td><td>146.51</td><td>109.31</td><td>315.7</td><td>117.2</td><td>58.0</td><td>55.1</td></tr>
<tr><td></td><td><MOR(7)|H|5w></td><td>1.755</td><td>0.137</td><td>3.56</td><td>146.99</td><td>109.21</td><td>316.9</td><td>117.6</td><td>56.2</td><td>53.6</td></tr>
<tr><td>T4</td><td>MOR(23)Na|0w</td><td>1.745</td><td>0.051</td><td>3.17</td><td>144.63</td><td>109.33</td><td>315.1</td><td>115.7</td><td>60.2</td><td>59.5</td></tr>
<tr><td></td><td>MOR(23)|Na|6w</td><td>1.742</td><td>0.123</td><td>3.15</td><td>142.91</td><td>109.19</td><td>314.7</td><td>114.3</td><td>62.3</td><td>61.6</td></tr>
<tr><td></td><td><MOR(23)|Na|6w></td><td>1.752</td><td>0.088</td><td>3.38</td><td>143.85</td><td>109.36</td><td>316.3</td><td>115.1</td><td>59.6</td><td>59.0</td></tr>
<tr><td></td><td><MOR(23)|Na|6w>*</td><td>1.752</td><td>0.086</td><td>3.37</td><td>144.84</td><td>109.38</td><td>316.4</td><td>115.9</td><td>58.7</td><td>57.0</td></tr>
<tr><td></td><td>MOR(7)|H|5w*</td><td>1.750</td><td>0.057</td><td>3.42</td><td>143.77</td><td>109.44</td><td>316.0</td><td>115.0</td><td>60.1</td><td>59.0</td></tr>
<tr><td></td><td><MOR(7)|H|5w></td><td>1.757</td><td>0.101</td><td>3.39</td><td>143.44</td><td>109.37</td><td>317.2</td><td>114.7</td><td>59.0</td><td>58.1</td></tr>
</table>

MOR(23)|Na|6w and MOR(23)|Na|6w* denote the states of the Na^{+} are partially solvated and completely solvated. In MOR(7)|H|5w, all three protons originating at T1, T3 and T4 are preferentially solvated in the static model(MOR(7)|H|5w*), and there is a competing configuration at +7 kJ mol^{-1} (MOR(7)|H|5w), in which T1 and T3 protons are unsolvated.

Table S7 ^{27}Al calculated NMR chemical shifts (δ) of CHA with Si/Al = 11 with optimized cell shape and atomic positions.

CHA	σ ($\langle\sigma\rangle$) (ppm)	δ ($\langle\delta\rangle$) (ppm)	Volume (Å^3)
bgc|0w[a]	494.33	60.94	810.90
**Na|0w[b]**	**494.46 (494.74)**	**60.79(60.48)**	**810.90**
Na|0w[c]	493.05	62.36	805.82
H(O1)|0w	497.71	57.19	811.79
H(O2)|0w	491.58	64.00	810.00
H(O3)|0w	493.15	62.24	811.42
**H(O4)|0w[d]**	**499.55 (498.41)**	**55.14 (56.41)**	**815.12**
**Na|1w**	**494.51**	**60.74**	**807.59**
H(O1)|1w	497.04	57.93	811.56
H(O2)|1w	493.13	62.27	800.52
H(O3)|1w	494.89	60.32	810.62
**H(O4)|1w**	**499.44**	**55.26**	**807.93**
**Na|5w[e]**	**493.44 (492.42)**	**61.93 (63.06)**	**796.35**
**Na|5w***	**494.84 (493.09)**	**60.37 (62.31)**	**796.35**
**H|5w***	**493.06 (493.09)**	**62.34 (62.32)**	**794.65**

[a] The framework negative charge is compensated with a homogeneous positive background charge; both the lattice parameters and atomic coordinates optimized with the CHA(11)|Na|0w model. [b] Na^+ located in the 6MR site. [c] Na^+ located in the 8MR site. [d] The most stable structure is the one with proton on O4; protons on O1, O2, and O3 are 7.7, 9.1, and 4.7 kJ/mol higher in energy, respectively (O1, O2, O3 and O4 are defined as in Fig. S1 following reference[1]). [e] Na|5w denotes the partially solvated Na^+, while Na|5w* and H|5w* denotes the fully solvated cations; Na|5w structure is 9.6 kJ/mol less stable than fully solvated Na|5w* structure. The rows in bold are the stable cases on which MD calculations were performed.

Table S8 Calculated ^{27}Al NMR chemical shifts (δ) and quadrupole coupling (C_q) of dehydrated Na-CHA with Si/Al = 47, 35 23 and 11.

	Na|0w				bgc (Vol= 802.05)		
Si/Al	σ(ppm)	δ (ppm)	C_q(MHz)	Volume (A^3)	σ (ppm)	δ (ppm)	C_q(MHz)
∞	-	-	-	802.05			
47	495.33	59.83	7.46	804.90	495.58	59.55	3.58
35	495.48	59.66	7.57	803.89	496.82	58.17	2.95
23	494.17	61.12	7.50	807.52	495.15	60.03	3.47
11	494.46	60.79	7.81	810.90	494.66	60.57	2.54
11(hex)[a]	494.01	61.30	7.74	811.57	493.87	61.45	2.55

a. This is the CHA(11)|Na|0w model with conventional hexagonal cell (as shown in Fig. S13).

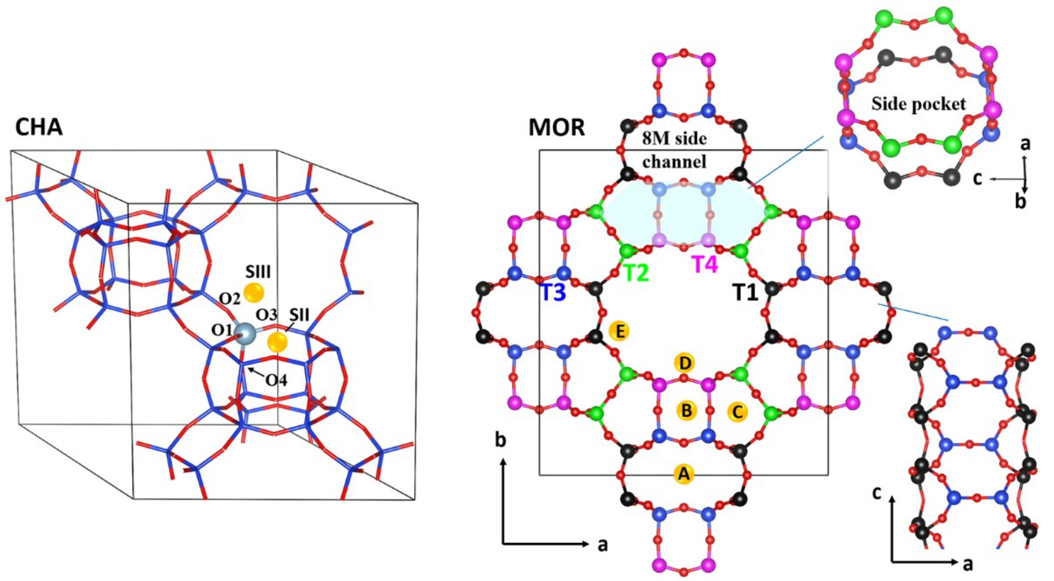

Fig. S2. The structure of zeolites CHA and MOR. There are 2 possible positions of Na^+ in CHA (left) and five in MOR (right).The yellow balls with number label denote possible Na^+ positions. CHA: SII is the 6MR position and SII 8MR position[2]; MOR: A, B, C, D and E are defined followed the reference.[3] Different T types of MOR are distinguished by colors: T1 black, T2 green, T3 blue, T4 pink.

Fig. S3. The detail of the models.

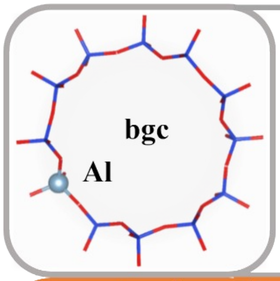

bgc|0w: background charge model

- Not explicitly considering any extra-framework cations
- The negative charge of the framework is compensated by a smeared background charge

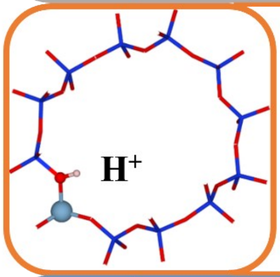

H|0w: dehydrated protonic model

- The negative charge of the framework is compensated by the proton bound to AlO_4
- The presence of proton on one of four oxygen atoms of AlO_4 tetrahedron results in large anisotropy on framework Al
- Different δ expected for each of for oxygen atoms

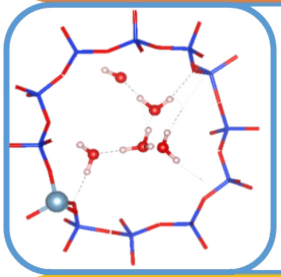

H|nw: hydrated protonic model

- The negative charge of the framework is compensated by the proton solvated in the vicinity of AlO_4
- Such situation occurs when there is sufficient void space with sufficient amount of water
- Charge compensation provided by the hydrogen bond network

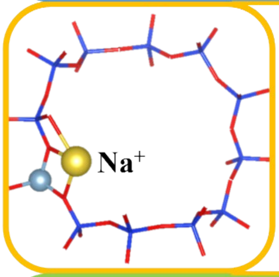

Na|0w: dehydrated Na^+ model

- The negative framework charge compensated by Na^+
- There may be more than one position of extra-framework Na^+ cation (depending on zeolite topology), each of them resulting in different δ

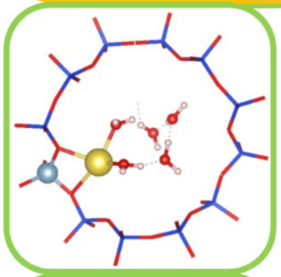

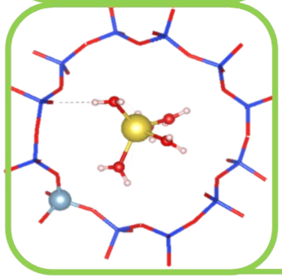

Na|nw: hydrated Na^+ model

- The negative framework charge compensated by Na^+ solvated in the vicinity of AlO_4
- Depending on the void space and amount of water in the vicinity of AlO_4 the Na^+ cation can be either partially or fully solvated
- Partial solvation: Na^+ stays coordinated to 2-3 framework oxygen atoms and it has 2-3 water molecules in the first coordination shell
- Full solvation: Na^+ has only water molecules in its first coordination shell and it is farther from AlO_4 tetrahedron

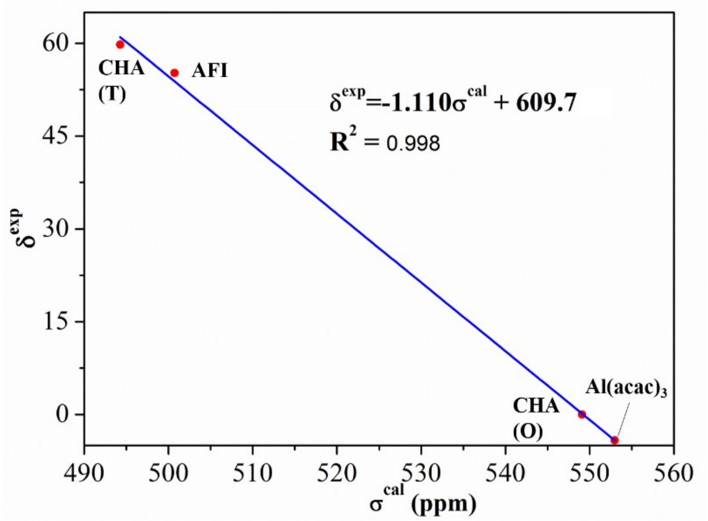

Fig. S4. A linear regression between the experimental chemical shift δ^{exp} and calculated chemical shielding σ^{cal} based on four experimental values: CHA(T) and AFI denotes tetrahedral framework Al in CHA and AFI, respectively;[4] CHA(O) denotes framework-associated octahedral Al sites;[5] $Al(acac)_3$ denotes octahedral Al in solid aluminum acetylacetone.[1]

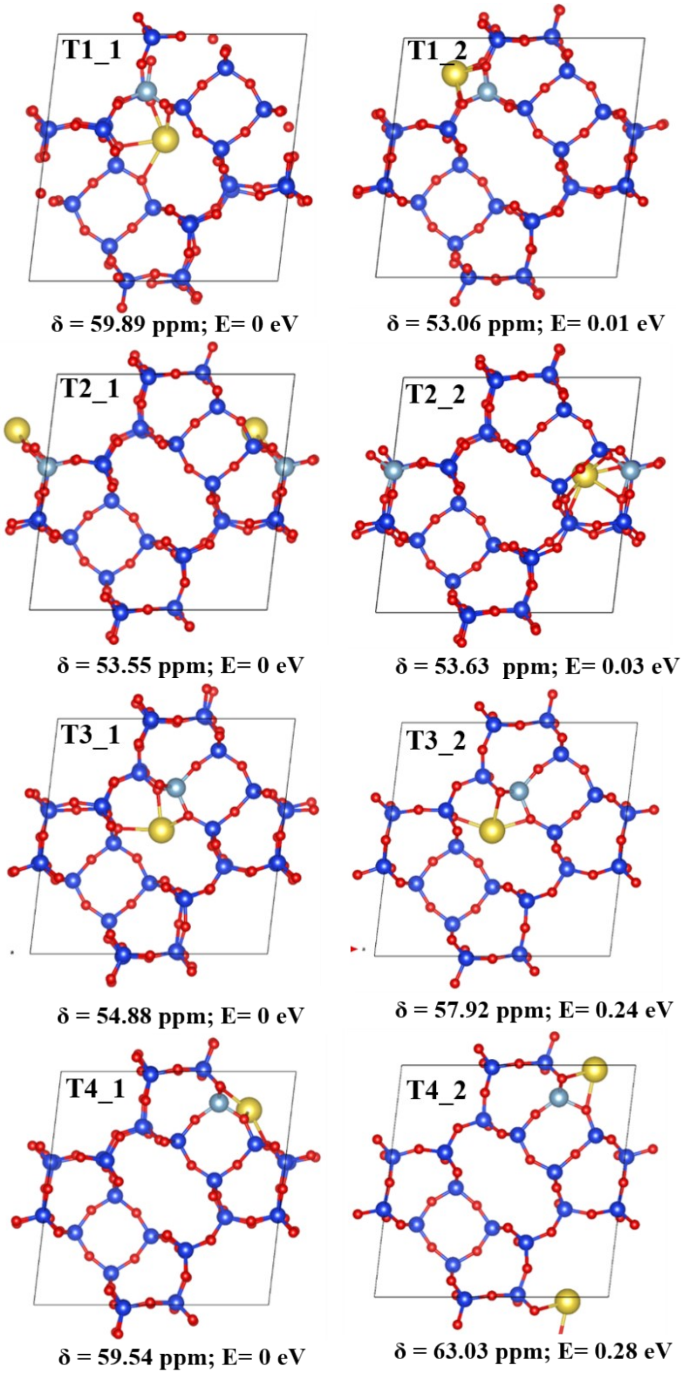

Fig. S5. Chemical shifts calculated for the two most stable MOR(23)|Na|0w local minimum configurations for each T site.

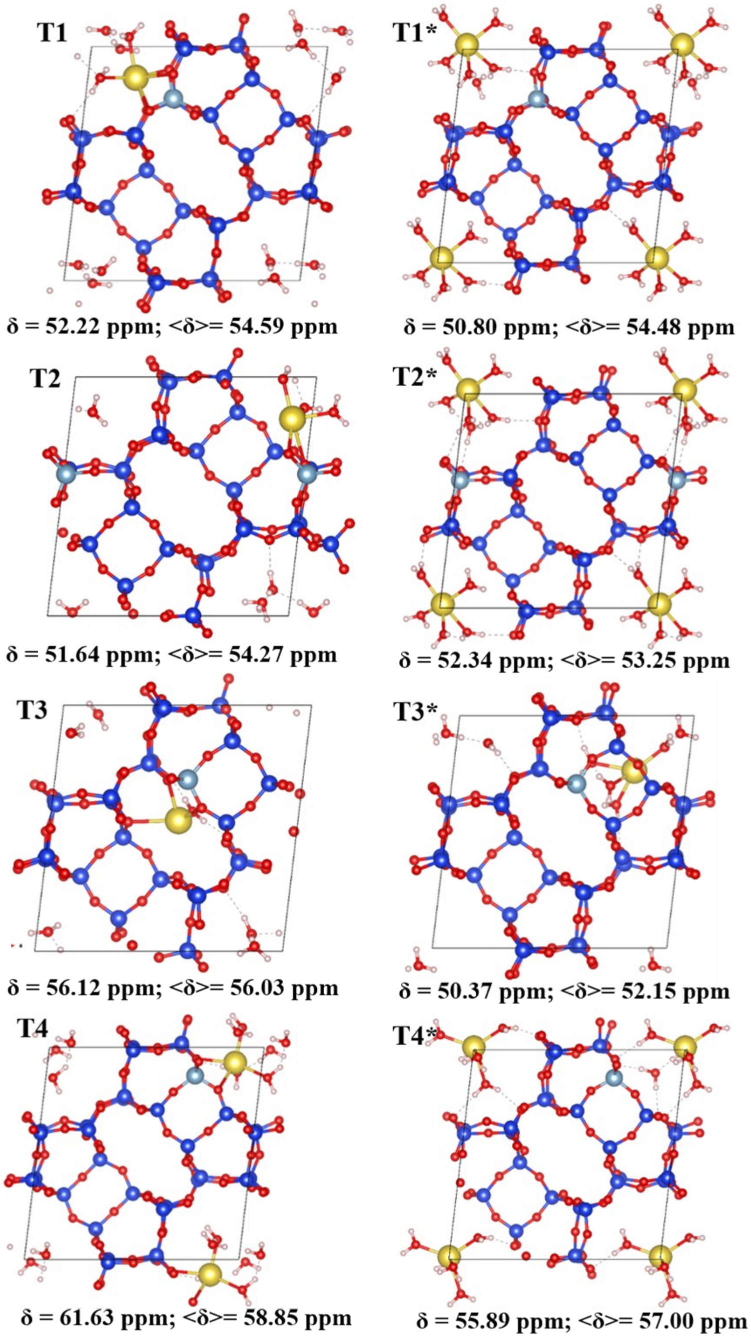

Fig. S6. Chemical shifts calculated for partially (left) and fully (right) solvated MOR(23)|Na|6w configurations for each T site. The configurations with solvated and fully solvated Na^+ are shown as Tn and Tn*, respectively (n=1-4). The partially solvated configurations are slightly more stable, with static energy differences of 6.8, 22.2, 28.0, and 23.2 kJ mol^{-1} to the fully solvated variants, for T1, T2, T3, and T4, respectively.

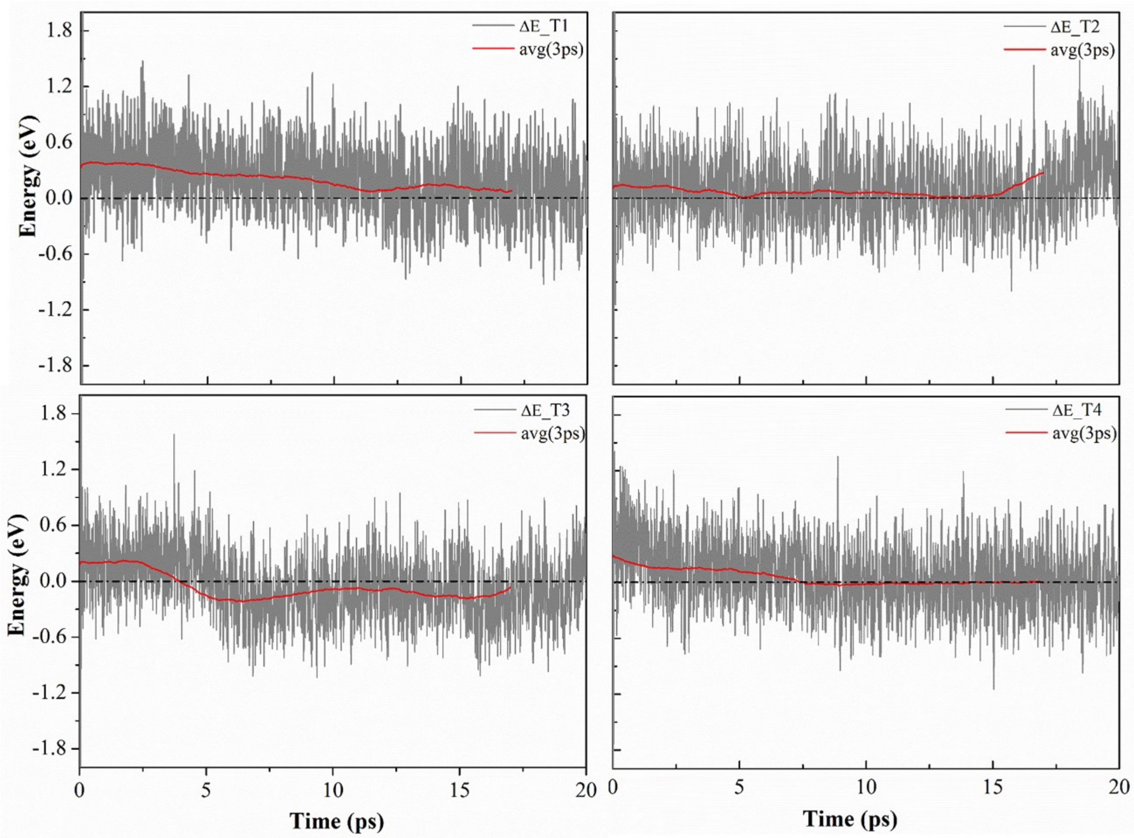

Fig. S7. The energy difference between the two trajectories of MOR(23)|Na|6w with partially and fully solvated Na. $\Delta E = E_{Tn}^{*} - \langle E_{Tn} \rangle$, $E_{Tn}(*)$ is the energy of each snapshot of trajectory of fully solvated case, $\langle E_{tn}(*) \rangle$ is the energy average of the partially solvated case. The red line is the moving average of 3 ps.

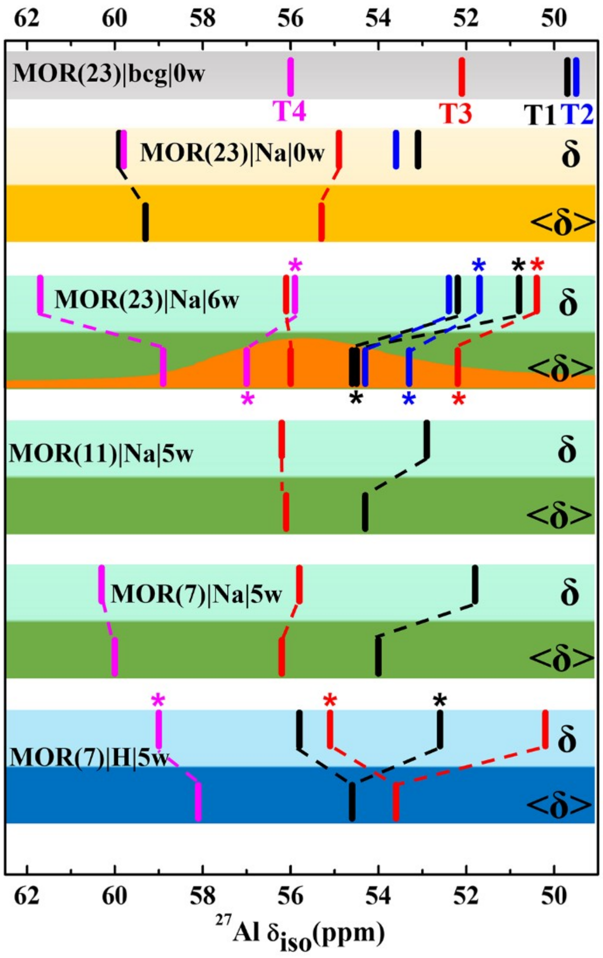

Fig. S8. Chemical shifts calculated for MOR (Si/Al from 23 to 7) of various models as in Fig. S3; Lighter color frames denote static shifts and darker color frames denote dynamic averaged results. In MOR(23)|Na|0w, only two cases with one T1 and T3 sites are used to verify that the dynamic process does not affect chemical shift in dehydrated condition. In MOR(11)|Na|5w, only one model is considered, with Al substituted into T1 and T3 sites. In MOR(7), Al is substituted into T1, T3 and T4. In MOR(11)|Na|5w, the Na^+ close to T1 is partially solvated, while the T3 cation is in the 8M side channel without solvation. In MOR(7)|Na|5w, the cations close to T1 and T4 are partially solvated, whereas the T3 cation is in the 8M side channel without solvation. In MOR(7)|H|5w, all three protons originating at T1, T3 and T4 are preferentially solvated in the static model, but there is a competing configuration at +7 kJ mol^{-1}, in which T1 and T3 protons are unsolvated. The protons at T1 and T3 rapidly fluctuate between solvated and unsolvated configurations along the MD trajectory. For T1, the proton is solvated for 56 % of snapshots along the trajectory, while for T3 it is solvated for 53 % of snapshots. Therefore, the structures are averages over two accessible states for both sites, with intermediate chemical shifts. Note: the asterisks are used to distinguish the cases with fully solvated Na^+ and solvated H^+.

CHA(11)|Na|5w

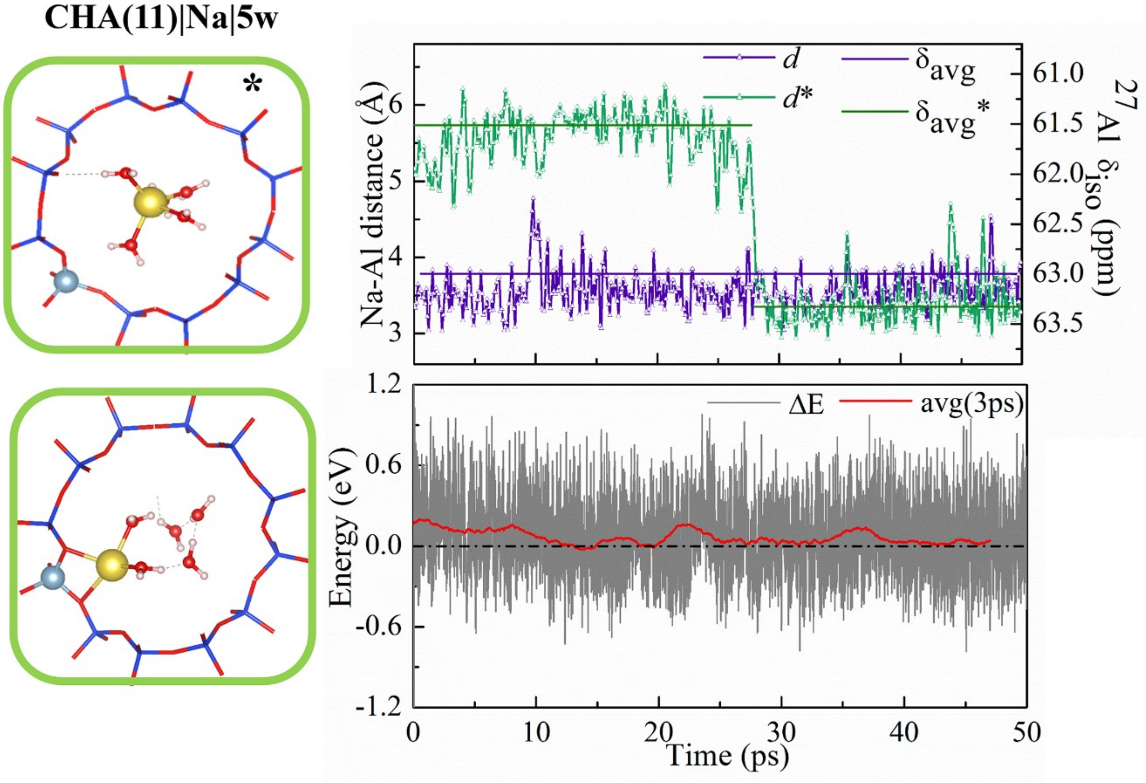

Fig. S9. The Al-Na distance and energy difference ΔE along the MD trajectory. ΔE is defined as the energy difference between fully solvated Na model and the averaged energy of the partially solvated model. The fully solvated Na^{+} cation is distinguished with an asterisk.

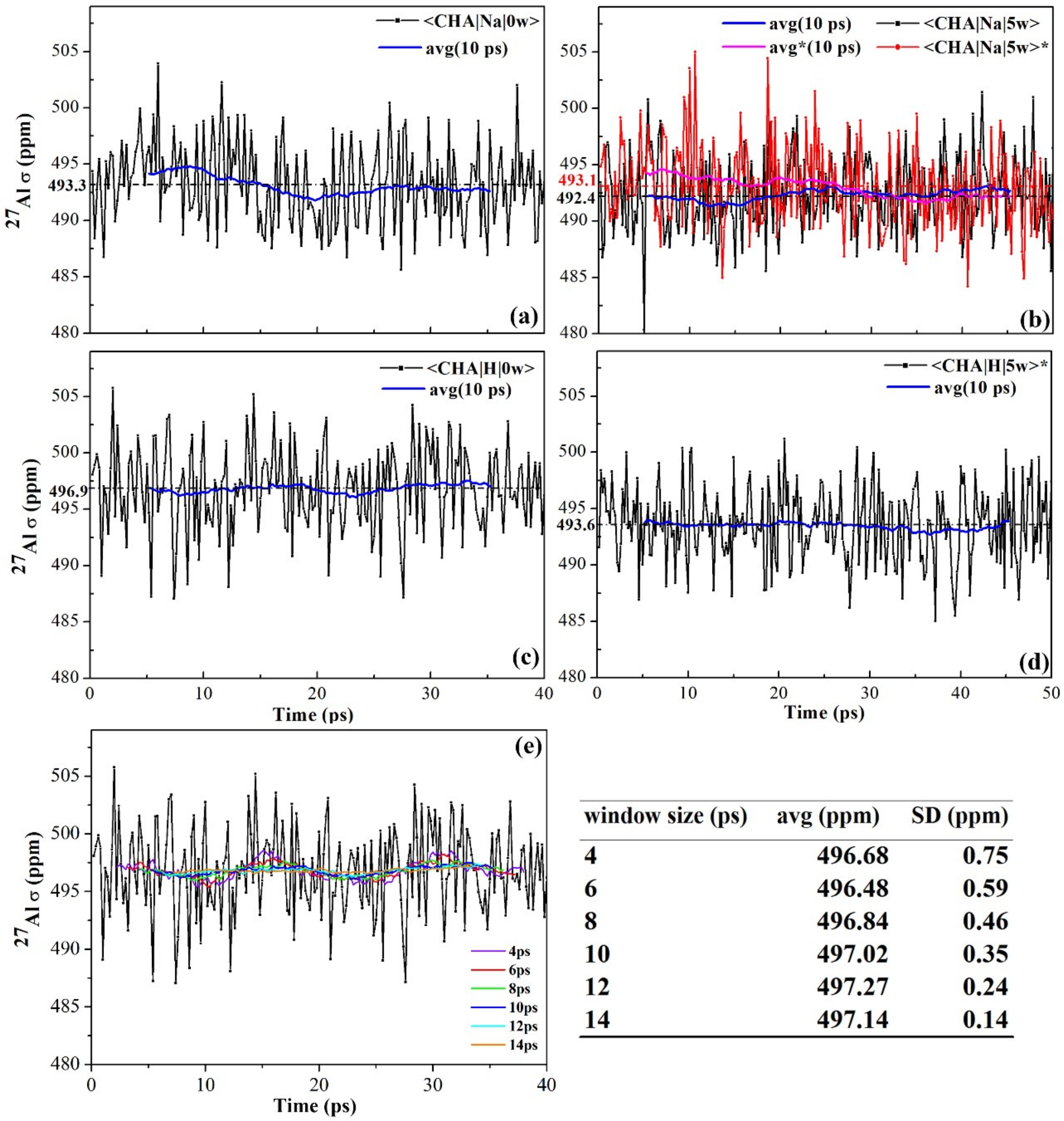

window size (ps)	avg (ppm)	SD (ppm)
4	496.68	0.75
6	496.48	0.59
8	496.84	0.46
10	497.02	0.35
12	497.27	0.24
14	497.14	0.14

Fig. S10. The ^{27}Al σ along the MD trajectory of (a) dehydrated Na-CHA, (b) hydrated Na-CHA (the asterisk is used to distinguish the case with the completely solvated Na^+ from the partially solvated Na^+ case), (c) dehydrated H-CHA, and (d) hydrated H-CHA. The dashed line is the average σ value along the trajectory. (e) The convergence of the prediction of average σ as a function of running average interval was tested on the dehydrated H-CHA data. All predicted average shieldings were within a 0.8 ppm envelope and standard deviations were well within the precision of the method (< 1.5 ppm) over the full range of intervals. The blue solid line denotes the running average of the data in black with 10 ps window, and the pink line is the average of the data in red.

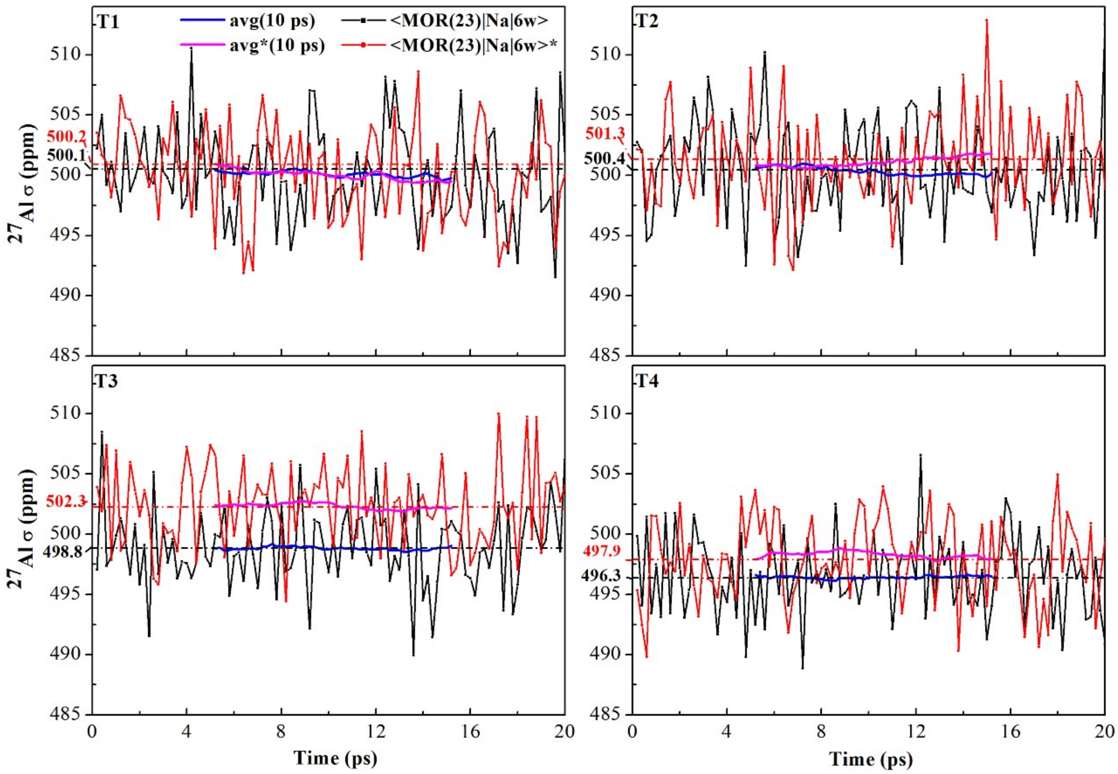

Fig. S11. The ^{27}Al σ along the MD trajectories of MOR(23)|Na|6w with different Al-substitution sites T1, T2, T3 and T4. The asterisk is used to distinguish the case with the fully solvated Na^+ (*) from the partially solvated Na^+ cases. The dashed line is the average σ value along the trajectory. The blue solid line denotes the running average of the data in black with 10 ps window size, and the pink line is the average of the data in red.

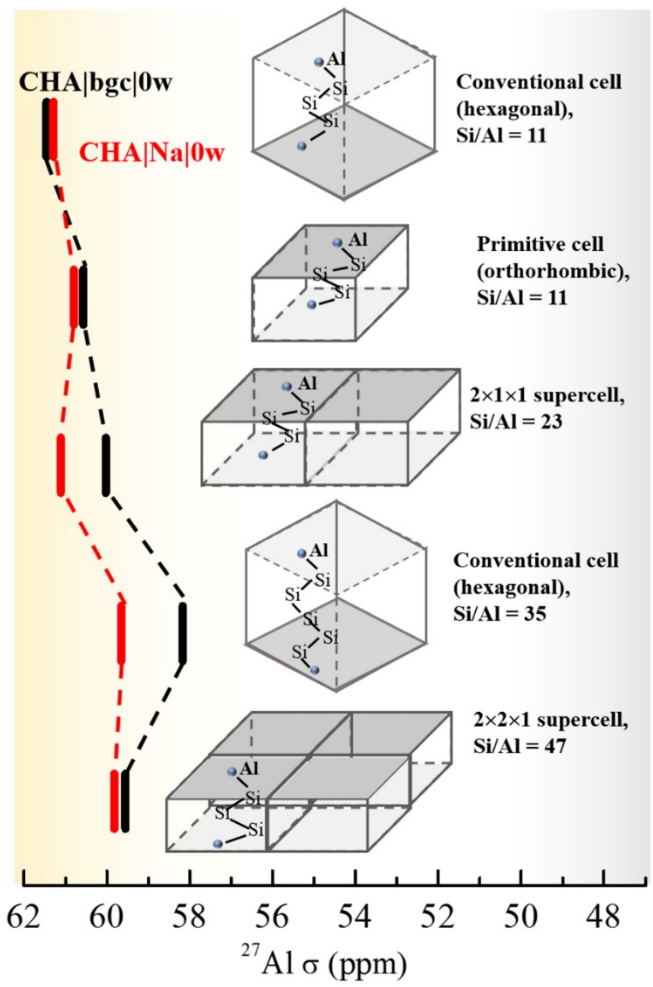

Fig. S12. The effect of the UC size and Si/Al ratio on calculated ^{27}Al chemical shifts, the data is also shown in the Table S8. Cell volumes are optimized for each supercell.

References

1. C. J. Heard, L. Grajciar, C. M. Rice, S. M. Pugh, P. Nachtigall, S. E. Ashbrook and R. E. Morriss, Fast room temperature lability of aluminosilicate zeolites, *Nature Communications*, 2019, **10**, 7.
2. L. J. Smith, H. Eckert and A. K. Cheetham, Site Preferences in the Mixed Cation Zeolite, Li,Na-Chabazite: A Combined Solid-State NMR and Neutron Diffraction Study, *Journal of the American Chemical Society*, 2000, **122**, 1700-1708.
3. W. J. Mortier and I. Z. A. S. Commission, *Compilation of Extra Framework Sites in Zeolites*, Butterworth Scientific Limited, 1982.
4. J. Holzinger, M. Nielsen, P. Beato, R. Y. Brogaard, C. Buono, M. Dyballa, H. Falsig, J. Skibsted and S. Svelle, Identification of Distinct Framework Aluminum Sites in Zeolite ZSM-23: A Combined Computational and Experimental ^{27}Al NMR Study, *The Journal of Physical Chemistry C*, 2019, **123**, 7831-7844.
5. M. Ravi, V. L. Sushkevich and J. A. van Bokhoven, On the location of Lewis acidic aluminum in zeolite mordenite and the role of framework-associated aluminum in mediating the switch between Brønsted and Lewis acidity, *Chemical Science*, 2021, **12**, 4094-4103.
